# Gene Expression Analysis in Three Posttraumatic Stress Disorder Cohorts Implicates Inflammation and Innate Immunity Pathways and Uncovers Shared Genetic Risk With Major Depressive Disorder

**DOI:** 10.3389/fnins.2021.678548

**Published:** 2021-07-29

**Authors:** Melanie E. Garrett, Xue Jun Qin, Divya Mehta, Michelle F. Dennis, Christine E. Marx, Gerald A. Grant, VA Mid-Atlantic MIRECC Workgroup, Mira Brancu, PTSD Initiative, Sarah McLeay, Injury and Traumatic Stress (INTRuST) Clinical Consortium, Psychiatric Genomics Consortium PTSD Group, Murray B. Stein, Nathan A. Kimbrel, Jean C. Beckham, Michael A. Hauser, Allison E. Ashley-Koch

**Affiliations:** ^1^Duke Molecular Physiology Institute, Duke University Medical Center, Durham, NC, United States; ^2^Queensland University of Technology, Centre for Genomics and Personalised Health, Faculty of Health, Institute of Health and Biomedical Innovation, Kelvin Grove, QLD, Australia; ^3^Durham Veterans Affairs Health Care System, Durham, NC, United States; ^4^VA Mid-Atlantic Mental Illness Research, Education, and Clinical Center, Durham, NC United States; ^5^Department of Psychiatry and Behavioral Sciences, Duke University School of Medicine, Durham, NC, United States; ^6^Department of Neurosurgery, Stanford University School of Medicine, Stanford, CA, United States; ^7^Gallipoli Medical Research Foundation, Greenslopes Private Hospital, Greenslopes, QLD, Australia; ^8^Faculty of Health, Queensland University of Technology, Kelvin Grove, QLD, Australia; ^9^Department of Psychiatry, School of Medicine, University of California, San Diego, La Jolla, CA, United States; ^10^Herbert Wertheim School of Public Health, University of California, San Diego, La Jolla, CA, United States; ^11^VA San Diego Healthcare System, San Diego, CA, United States

**Keywords:** posttraumatic stress disorder, gene expression, major depressive disorder, quantitative trait loci, multi-ethnic, meta-analysis

## Abstract

Posttraumatic stress disorder (PTSD) is a complex psychiatric disorder that can develop following exposure to traumatic events. The Psychiatric Genomics Consortium PTSD group (PGC-PTSD) has collected over 20,000 multi-ethnic PTSD cases and controls and has identified both genetic and epigenetic factors associated with PTSD risk. To further investigate biological correlates of PTSD risk, we examined three PGC-PTSD cohorts comprising 977 subjects to identify differentially expressed genes among PTSD cases and controls. Whole blood gene expression was quantified with the HumanHT-12 v4 Expression BeadChip for 726 OEF/OIF veterans from the Veterans Affairs (VA) Mental Illness Research Education and Clinical Center (MIRECC), 155 samples from the Injury and Traumatic Stress (INTRuST) Clinical Consortium, and 96 Australian Vietnam War veterans. Differential gene expression analysis was performed in each cohort separately followed by meta-analysis. In the largest cohort, we performed co-expression analysis to identify modules of genes that are associated with PTSD and MDD. We then conducted expression quantitative trait loci (eQTL) analysis and assessed the presence of eQTL interactions involving PTSD and major depressive disorder (MDD). Finally, we utilized PTSD and MDD GWAS summary statistics to identify regions that colocalize with eQTLs. Although not surpassing correction for multiple testing, the most differentially expressed genes in meta-analysis were interleukin-1 beta (*IL1B*), a pro-inflammatory cytokine previously associated with PTSD, and integrin-linked kinase (*ILK*), which is highly expressed in brain and can rescue dysregulated hippocampal neurogenesis and memory deficits. Pathway analysis revealed enrichment of toll-like receptor (TLR) and interleukin-1 receptor genes, which are integral to cellular innate immune response. Co-expression analysis identified four modules of genes associated with PTSD, two of which are also associated with MDD, demonstrating common biological pathways underlying the two conditions. Lastly, we identified four genes (*UBA7*, *HLA-F*, *HSPA1B*, and *RERE*) with high probability of a shared causal eQTL variant with PTSD and/or MDD GWAS variants, thereby providing a potential mechanism by which the GWAS variant contributes to disease risk. In summary, we provide additional evidence for genes and pathways previously reported and identified plausible novel candidates for PTSD. These data provide further insight into genetic factors and pathways involved in PTSD, as well as potential regions of pleiotropy between PTSD and MDD.

## Introduction

Posttraumatic stress disorder (PTSD) is a common psychiatric disorder that can occur following exposure to traumatic events. It is characterized by re-experiencing symptoms, avoidance, and persistent hyperarousal. While 7–8% of adults in the United States will experience PTSD over the course of their lifetime (Kessler et al., 2005; Roberts et al., 2011; Kilpatrick et al., 2013), rates are much higher among military veterans. The prevalence of PTSD in veterans returning from Iraq/Afghanistan is estimated to be 23% (Fulton et al., 2015), whereas an estimated 30% of Vietnam veterans have experienced lifetime PTSD (Kulka et al., 1990). Individuals with PTSD are at increased risk for many other comorbid conditions including major depressive disorder (MDD) (Cougle et al., 2010; Rytwinski et al., 2013; Flory and Yehuda, 2015; Ben Barnes et al., 2018), substance abuse disorder (SUD) (Petrakis et al., 2011; Seal et al., 2011; Blanco et al., 2013; Stein et al., 2017; Teeters et al., 2017), sleep disorders (Pigeon et al., 2013; Lind et al., 2020), and cardiovascular disease (Pollard et al., 2016; Koenen et al., 2017). Individuals with PTSD are also at increased risk for suicidal behaviors, particularly if they experience comorbid MDD (Oquendo et al., 2005; Panagioti et al., 2012; Kimbrel et al., 2016; Livingston et al., 2020; Ursano et al., 2020). For example, Kimbrel et al. (2016) found that among Iraq/Afghanistan-era war veterans, comorbid PTSD-depression was a robust prospective predictor of future suicide attempts over a 12-month period, even after controlling for the effects of sex, age, race, sexual orientation, and lifetime history of suicide attempts. Given the rate of death by suicide among veterans is approximately 1.5 times the rate among non-veteran adults (Department of Veterans Affairs, Office of Mental Health & Suicide Prevention, 2020^1^), it is clear that an improved understanding of the etiology of PTSD and depression is of utmost importance.

While most people experience at least one traumatic event in their life, only some will subsequently develop PTSD (Benjet et al., 2016; Liu et al., 2017), suggesting that a heritable component to risk for PTSD exists. Based on family and twin studies, genetic susceptibility accounts for an estimated 30–70% of the variance in PTSD risk (True et al., 1993; Yehuda et al., 2001; Stein et al., 2002; Wolf et al., 2014). There is also evidence for a shared heritable influence on PTSD and MDD (Sartor et al., 2012). Utilizing over 20,000 individuals from 11 studies, the Psychiatric Genomics Consortium-Posttraumatic Stress Disorder group (PGC-PTSD) recently reported a single nucleotide polymorphism (SNP) heritability estimate of 29% for European-American females and found evidence for overlapping genetic risk between PTSD and schizophrenia, bipolar disorder, and MDD (Duncan et al., 2018). Even with large sample sizes, it has been difficult to identify robust regions of association with PTSD, illustrating the complex and multi-genic nature of the disorder. Recent GWAS reports from the PGC-PTSD and Million Veteran Program (MVP) have had greater success in identifying PTSD-associated loci when utilizing quantitative symptom severity and symptom subdomains instead of PTSD diagnosis (Maihofer et al., submitted; Stein et al., 2021). Nonetheless, the biological mechanisms underlying these largely non-coding regions of association have not yet been elucidated.

Gene expression profiling has been widely used to identify genes and pathways that are associated with specific biological processes and to study related molecular mechanisms. A recent transcriptome-wide study in prefrontal cortex (PFC) of post-mortem human brains identified co-regulated gene networks that are altered in PTSD, including differences between men and women which could help explain the increased prevalence of PTSD among women (Girgenti et al., 2021). However, due to the extremely limited availability of human brain tissue from PTSD-affected donors, the majority of studies have focused on gene expression in human peripheral blood samples. Although this is a limitation, evidence from both human and animal studies indicate it is likely that changes in the blood transcriptome reflect at least some changes occurring in the brain. For example, inflammation in the periphery has been associated with global brain transcriptome changes in mouse (Thomson et al., 2014). In humans, Huckins et al. (2020) showed that genetically regulated transcriptomic changes in the brain correlate with measured gene expression changes in peripheral blood.

Many previous gene expression studies of peripheral blood in PTSD patients and controls have reported that alterations in immune and inflammatory response pathways are important in PTSD pathogenesis, specifically the hypothalamic-pituitary-adrenal (HPA) axis and glucocorticoid (GC) function (Segman et al., 2005). For example, FK506 binding protein 5 (*FKBP5*), a GC receptor inhibitor, is downregulated in PTSD cases (Yehuda et al., 2009). This finding, along with other genes in the GC receptor signaling pathway, has been widely replicated across various trauma types (Mehta et al., 2011, 2018; Sarapas et al., 2011; van Zuiden et al., 2012; Logue et al., 2015; Kuan et al., 2017). Others have reported differentially expressed genes in pathways enriched for innate immunity and inflammatory response (Zieker et al., 2007; Neylan et al., 2011; Breen et al., 2015, 2018; Guardado et al., 2016; Rusch et al., 2019). Additionally, decreased interleukin 1A (*IL1A*) expression was observed in the dorsolateral prefrontal cortex (dlPFC) of post-mortem PTSD brains (Morrison et al., 2019), a finding that is concordant with reports of altered cytokine expression in peripheral blood of PTSD patients (Zieker et al., 2007; Mehta et al., 2011; Breen et al., 2018).

Taken together, the findings described above suggest that altered transcripts in peripheral blood can still provide meaningful insights into the pathophysiology of PTSD. However, most of the studies to date have relied upon small sample sizes and did not include subjects from diverse ancestry groups. Accordingly, the objective of the current study was to conduct the largest meta-analysis of differential PTSD gene expression to date (*n* = 977), including 383 PTSD cases and 594 trauma-exposed controls from three multi-ethnic cohorts. Additional analyses aimed at uncovering genetic regulators of expression and modules of co-expression were performed in the largest cohort (*n* = 726). Finally, we sought to explain previously reported PTSD and MDD GWAS regions by alterations in gene expression.

## Materials and Methods

### Study Participants

Nine hundred seventy-seven subjects with available gene expression, genotype, and clinical phenotype data were selected from three independent PGC-PTSD cohorts: veterans returning from Iraq/Afghanistan from the Veterans Affairs (VA) VISN-6 Mid-Atlantic Mental Illness Research Education and Clinical Center (MIRECC) as well as community civilians and all-era veterans enrolled in other trauma research studies at the Durham VA Health Care System and Duke University Medical Center (MIRECC/Duke; *n* = 726), the Injury and Traumatic Stress (INTRuST) Clinical Consortium (*n* = 155), and Australian Vietnam War veterans (GMFR-QUT; *n* = 96), which have been described previously (Ashley-Koch et al., 2015; McLeay et al., 2017; Akosile et al., 2018; Bomyea et al., 2019, 2020).

Posttraumatic stress disorder diagnosis for the MIRECC/Duke cohort was determined using either the Structured Clinical Interview for DSM-IV Disorders (SCID; First et al., 1994) or the Clinician-Administered PTSD Scale (CAPS; Blake et al., 1995), whereas PTSD severity was measured using the Davidson Trauma Scale (DTS; Davidson et al., 1997). For the INTRuST consortium, PTSD diagnosis was determined using either the PTSD Checklist-Civilian Version (PCL-C; Weathers et al., 1993), the CAPS (Blake et al., 1995), or the MINI International Neuropsychiatric Interview 6.0.0 (MINI; Sheehan et al., 1998), and for the GMRF-QUT cohort, PTSD diagnosis was determined with the CAPS-5 (Weathers et al., 2001). While the CAPS and SCID are considered gold standard interviews for PTSD, the MINI (compared to the SCID) has demonstrated a sensitivity of 0.85 and specificity of 0.96 for PTSD (Sheehan et al., 1998). Diagnosis based on a PCL cutoff of 50 (as used in the INTRuST cohort) has a sensitivity of 0.52 and a specificity of 0.94 for PTSD compared to the CAPS (Yeager et al., 2007). MDD was assessed in the MIRECC/Duke cohort using the SCID (First et al., 1994), whereas in the INTRuST consortium, depression was assessed using the Patient Health Questionnaire-9 (PHQ9; Kroenke et al., 2001) and scores ≥ 15 indicated the subject had MDD. For the GMRF-QUT cohort, MDD was assessed with the MINI (Sheehan et al., 1998). While the SCID is a gold standard interview for MDD, the PHQ-9 has established reliability and validity to detect a high probability of MDD. Using the Mental Health Professional Validation Interview as the criterion standard, a PHQ-9 score ≥ 15 (as used in the INTRuST consortium) had a sensitivity of 0.68 and a specificity of 0.95 for MDD (Kroenke et al., 2001). The MINI, which was used in the GMRF-QUT cohort, demonstrated a sensitivity of 0.96 and a specificity of 0.88 compared to the SCID (Sheehan et al., 1998). Smoking status for participants in each cohort was determined using study-specific questionnaires. Each study received approval from their respective ethics committee or institutional review board (IRB), and informed consent was obtained from each study participant prior to data collection.

### Gene Expression Microarrays

Gene expression data was generated in the three cohorts separately using HumanHT-12 v4 Expression BeadChips (Illumina Inc., San Diego, CA, United States), which capture 47,231 expression probes. For the MIRECC/Duke and INTRuST samples, whole blood was collected in PAXgene blood RNA tubes and incubated at room temperature overnight before being transferred to −20°C for 24 h and finally stored at −80°C. Total RNA was extracted using the PAXgene Blood RNA System Kit following manufacturer’s guidelines (Qiagen, Germantown, MD, United States). Purified RNA was analyzed for integrity (RIN) using the Agilent Bioanalyzer 2100 with Agilent RNA 6000 LabChip kits (Agilent, Santa Clara, CA, United States) and only samples with RIN ≥ 6 were included in downstream analyses. All RNA samples were processed using the Ambion GLOBINclear-Human Globin mRNA Removal Kit to deplete alpha and beta globin mRNA and to increase sensitivity of gene detection (Life Technologies, Foster City, CA, United States). The enriched RNA was amplified and biotin-labeled using the Illumina TotalPrep Amplification Kit before hybridization to the microarrays. Details regarding the generation of gene expression data for the GMRF-QUT cohort have been described previously (Mehta et al., 2017, 2018).

### Single Nucleotide Polymorphism Genotyping and Imputation

Information on SNP genotyping and imputation for all three cohorts have been previously described (Ashley-Koch et al., 2015; Mehta et al., 2017; Nievergelt et al., 2019). Briefly, DNA was hybridized to three different bead chips in three different batches for the MIRECC/Duke samples: HumanHap650 BeadChip, Human1M-Duo BeadChip, and HumanOmni2.5 BeadChip (Illumina, San Diego, CA, United States). Resulting genotypes were then merged and imputed using a global reference panel from 1000Genomes (1000 Genomes Project Consortium et al., 2015). For GMRF-QUT, genotypes were obtained from the Infinium PsychArray (Illumina Inc., San Diego, CA, United States) and were imputed with the 1000Genomes phase 3 reference panel on the Michigan Imputation Server (imputationserver.sph.umich.edu). INTRuST samples were genotyped using the Infinium PsychArray (Illumina Inc., San Diego, CA, United States) and were imputed with IMPUTE2 (Howie et al., 2009) using the 1000Genomes phase 3 reference panel. Principal component analysis (PCA) of SNPs from each cohort merged with hapmap3 data (International HapMap 3 Consortium et al., 2010) was performed and resulting PCs were plotted to visualize the population substructure present within each cohort using R.

### Statistical Analysis

Sample characteristics were assessed for differences by cohort using SAS v9.4 (SAS Institute, Cary, NC, United States). Raw gene expression data was processed and quality control measures were taken for each cohort separately in R. For the MIRECC/Duke and INTRuST cohorts, probes below the detection threshold (detection *p* > 0.05) for more than half of the samples were removed. Background adjustment, log2 transformation, and quantile normalization were performed using limma (Ritchie et al., 2015). PCA was performed to check for batch effects, which were subsequently corrected using ComBat (Johnson et al., 2007). For GMRF-QUT, data were transformed and normalized using the variance stabilizing normalization (Huber et al., 2002), and probes detected in ≥5% of samples (detection *p*-value < 0.05) were retained for analysis (Mehta et al., 2017). For all cohorts, the proportion of monocytes and lymphocytes for each sample was estimated using an approach described in Logue et al. (2015). The following number of probes were available for analysis in each cohort: 17,776 for MIRECC/Duke, 13,586 for INTRuST, and 12,613 for GMRF-QUT. Differential gene expression analysis for PTSD was performed with a common pipeline in each cohort separately controlling for age, principal components (PCs), sex (if applicable), smoking status, and cell proportions using limma, followed by meta-analysis of the 11,502 overlapping probes using the inverse normal method (Marot et al., 2009; Ratanatharathorn et al., 2017). Inflation and bias were controlled using the empirical null distribution (van Iterson et al., 2017) and multiple testing was corrected using false discovery rate (FDR; Storey and Tibshirani, 2003). Pathway analysis was performed using DAVID (Huang da et al., 2009a,b). Unique genes corresponding to gene expression probes with meta-analysis *p* < 0.05 were used as the gene list of interest and all unique genes corresponding to the 11,502 gene expression probes common to all three cohorts were included as the background gene list. Finally, a volcano plot depicting the differential expression meta-analysis *p*-values and log fold changes obtained from the MIRECC/Duke cohort, which is the largest of the three cohorts included, was generated using R.

We next performed an expression quantitative trait loci (eQTL) analysis in the MIRECC/Duke subjects, stratified by ethnicity, using fastQTL (Ongen et al., 2016). Specifically, each gene expression probe was regressed on SNP genotypes within a 1MB window, using an additive genetic model and controlling for 10 PCs from PCA of normalized gene expression values and 10 PCs from PCA of SNP data. Only the most significant SNP for each probe was retained. Using the most significant SNP for each probe, we then tested for interactions with current PTSD status, controlling for the same 20 PCs listed above, in PLINK. Likewise, we interrogated SNP by current MDD interactions using the same approach.

Utilizing summary statistics from the most recent PGC-PTSD GWAS (Maihofer et al., submitted) and MDD GWAS (Wray et al., 2018), we tested for colocalization with eQTLs identified in 309 non-Hispanic white (NHW) MIRECC/Duke subjects. There were 20 GWAS-associated regions for PTSD and 44 regions for MDD. All eQTLs involving a gene which resides in these 64 regions were interrogated for evidence of a shared causal variant with SNPs from the PTSD and MDD GWAS studies using coloc (Giambartolomei et al., 2014), a Bayesian method that investigates five possible hypotheses: no association (H_0_), association with PTSD (MDD) but not gene expression (H_1_), association with gene expression but not PTSD (MDD) (H_2_), two distinct causal variants, each associated with one trait (H_3_), or a shared causal variant that is associated with both PTSD (MDD) and gene expression (H_4_). Regions with posterior probability for H_4_ (PP_*H4*_) > 0.90 were deemed to co-localize.

To identify modules of co-regulated gene expression, weighted correlation network analysis was performed with WGCNA (Zhang and Horvath, 2005; Langfelder and Horvath, 2008) in all MIRECC/Duke subjects (*n* = 726) and subsequently in 309 NHW MIRECC/Duke subjects and 417 non-Hispanic black (NHB) MIRECC/Duke subjects separately. Probes missing for > 15% of samples were removed. For genes with > 1 expression probe, only the most variable probe was retained. Finally, the distribution of probe variability was inspected and the 50% most variable probes were included in WGCNA (*n* = 5,070 probes for all subjects, *n* = 5,187 probes for NHW subjects, and *n* = 4,993 probes for NHB subjects). A soft-threshold parameter was chosen to approximate scale-free topology, modules were detected with a minimum size = 30, and resulting modules were merged when the correlation coefficient was ≥ 0.75. Logistic regression was used to test for association between each module eigengene and current PTSD, current MDD, and current smoking status, controlling for age, sex, PCs, and cell type estimates. Current smoking status was included as a covariate in the models for current PTSD and current MDD. Total DTS score was also tested for association with each module eigengene controlling for the same covariates and assuming a zero-inflated negative binomial distribution. Pathway analysis using DAVID was performed on resulting co-expression modules with module members as the gene list of interest and all genes input into WGCNA (described above) as the background gene list.

## Results

A description of sample characteristics by cohort is shown in Table 1. Subjects from GMRF-QUT were significantly older, exclusively male, more likely to be non-smokers, and had a higher percentage of PTSD compared to those from MIRECC/Duke or INTRuST. The percentage of MDD in the MIRECC/Duke cohort was higher compared to INTRuST and GMRF-QUT. PTSD and MDD were significantly correlated in all three cohorts (*p* ≤ 0.0001) and Cramer’s V between PTSD and MDD was highest among INTRuST subjects (V_*GMRF–QUT*_ = 0.38, V_*MIRECC/Duke*_ = 0.43, V_*INTRuST*_ = 0.53). The MIRECC/Duke subset utilized in this analysis was comprised of 309 NHW and 417 NHB subjects. PCA plots of each cohort overlaid with HapMap3 samples show that the GMRF-QUT samples were primarily of European descent, whereas the INTRuST samples were multi-ethnic (Supplementary Figures 1–3).

**TABLE 1:** Sample characteristics by cohort.

	MIRECC/Duke (*n* = 726)	INTRuST (*n* = 155)	GMRF-QUT (*n* = 96)	*p*-value
Mean age (SD)	37.7 (10.1)	32.5 (11.0)	68.7 (4.4)	<0.0001
% Female	22.9%	40.0%	0.0%	<0.0001
% PTSD	39.4%	31.6%	50.0%	0.0146
% MDD	26.1%	11.2%	12.5%	<0.0001
% Smoking	28.4%	19.1%	6.3%	<0.0001

**FIGURE 1:**
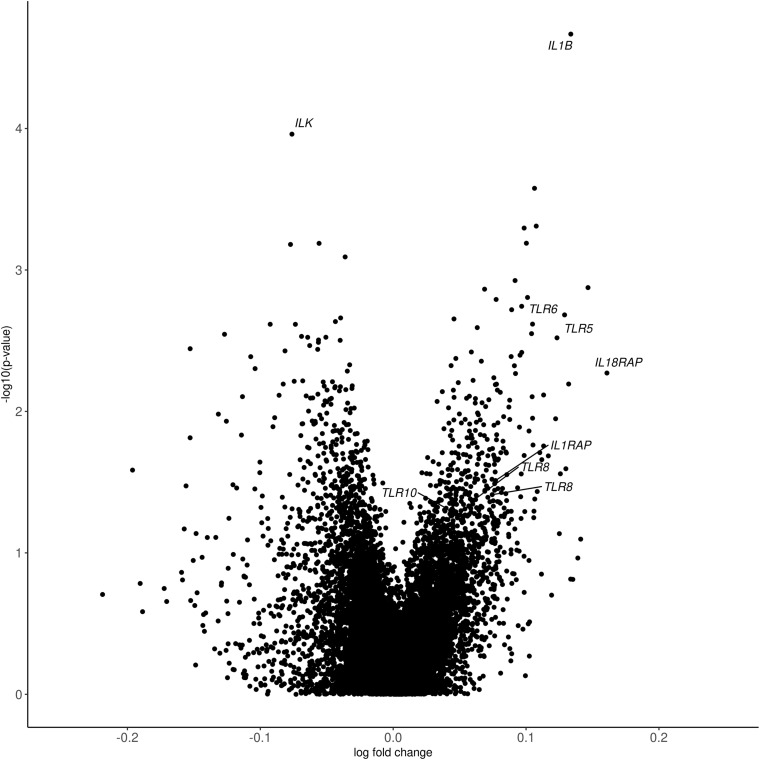
Volcano plot depicting differential gene expression *p*-values from meta-analysis and log fold change from the MIRECC/Duke cohort. In each cohort, differences in gene expression between PTSD cases and controls were interrogated controlling for age, PCs, sex (except in GMRF-QUT, which was exclusively male), smoking status, and cell proportions. The most significant genes (*IL1B* and *ILK*) and those from the enriched Toll-interleukin 1 resistance pathway (SM00255:TIR; *TLR5*, *TLR6*, *TLR8*, *TLR10*, *IL1RAP*, and *IL18RAP*) are labeled.

Despite these differences in cohort characteristics, meta-analysis revealed 558 genes that were differentially expressed between PTSD cases and controls (*p* < 0.05, Supplementary Table 1). Inflation and bias were sufficiently controlled (λ_*GC*_ = 1.1). Meta-analysis *p*-values and effect sizes from the largest cohort (MIRECC/Duke) are depicted in Figure 1. For nominally significant probes (*p* < 0.05), the direction of effect is concordant 91% of the time for MIRECC/Duke and INTRuST, and is concordant 72% of the time for MIRECC/Duke and GMRF-QUT. Although not surpassing correction for multiple testing, the most significant genes were interleukin-1 beta (*IL1B*; *p* = 2.15 × 10^–5^), and integrin-linked kinase (*ILK*; *p* = 1.10 × 10^–4^). PTSD cases displayed higher expression of *IL1B* compared to controls, whereas *ILK* expression was lower in PTSD cases compared to controls. Pathway analysis of all nominally associated genes (*p* < 0.05) revealed an enrichment of genes involved in Toll-interleukin 1 resistance (SM00255:TIR; *p* = 7.01 × 10^–4^), including *TLR5*, *TLR6*, *TLR8*, *TLR10*, *IL1RAP*, and *IL18RAP*, which represent seven unique expression probes that were all upregulated in PTSD cases.

Quantitative trait loci analysis identified many SNPs associated with expression levels: 2,216 in the NHW MIRECC/Duke subset and 1018 in the NHB subset (FDR *q* < 0.05). Subsequently, we performed an interaction analysis and identified several SNPs that were associated with gene expression at levels surpassing correction for multiple testing, but only in those with either PTSD or MDD. In the NHB subset, rs28842268 was associated with ILMN_1764090 in *AK3L1* (*AK4*), but only among those with PTSD (*p* = 7.9 × 10^–6^, *q* = 0.0473, Supplementary Figure 4A). Likewise, in the NHB subset, rs11823726 was associated with ILMN_1759789 in *KAT5*, but only among those with PTSD (*p* = 8.24 × 10^–6^, *q* = 0.0473, Supplementary Figure 4B). In the NHW subset, we observed two eQTLs that were significant only in those with MDD. rs28536123 was associated with ILMN_2134888 in *TUBE1* (*p* = 3.43 × 10^–9^, *q* = 4.25 × 10^–5^, Supplementary Figure 4C) and rs687562 was associated with ILMN_1872122 in *LCOR* (*p* = 1.63 × 10^–6^, *q* = 0.0101, Supplementary Figure 4D), but only among those with MDD. These results should be viewed as preliminary due to small sample size and require replication in independent cohorts.

Next, Bayesian colocalization analysis was used to detect regions with high probability of a shared causal variant for PTSD or MDD and gene expression (Table 2). One eQTL in Ubiquitin Like Modifier Activating Enzyme 7 (*UBA7*; PP_*H4*_ = 0.9604) colocalized with PTSD associated variants, whereas two eQTLs were found to colocalize with MDD associated variants: one in *HLA-F* (PP_*H4*_ = 0.9844) and one in *HSPA1B* (PP_*H4*_ = 0.9509). Several other regions displayed suggestive evidence for colocalization, including two separate eQTLs in *RERE* (PP_*H4*_ = 0.8757 and 0.8488) with MDD associated variants. Interestingly, the eQTL in *UBA7* deemed to colocalize with PTSD associated variants also showed suggestive evidence for colocalization with MDD associated variants (PP_*H4*_ = 0.8178).

**TABLE 2:** Colocalization of eQTLs in NHW MIRECC/Duke subset and either PTSD or MDD associated GWAS regions (PP_*H4*_ > 0.8).

Gene expression probe	Gene	PTSD GWAS region (Maihofer et al., submitted)	MDD GWAS regions (Wray et al., 2018)	Summary statistics used for analysis	N SNPs tested	Posterior probability of shared causal variant (PP_*H4*_)
ILMN_1794612	*UBA7*	chr3:49734229-50209053		PTSD	1,254	0.9604
ILMN_1762861	*HLA-F*		chr6:27738000-32848000	MDD	628	0.9844
ILMN_1660436	*HSPA1B*		chr6:27738000-32848000	MDD	1,574	0.9509
ILMN_1802380	*RERE*		chr1:8390000-8895000	MDD	1,341	0.8757
ILMN_2327795	*RERE*		chr1:8390000-8895000	MDD	1,341	0.8488
ILMN_1726288	*TMEM106B*		chr7:12154000-12381000	MDD	4,362	0.8426
ILMN_3238859	*FAM120AOS*	chr9:96181075-96381916		MDD	2,907	0.8276
ILMN_1738239	*RBM6*	chr3:49734229-50209053		MDD	1,538	0.8253
ILMN_1794612	*UBA7*	chr3:49734229-50209053		MDD	1,363	0.8178
						

**FIGURE 2:**
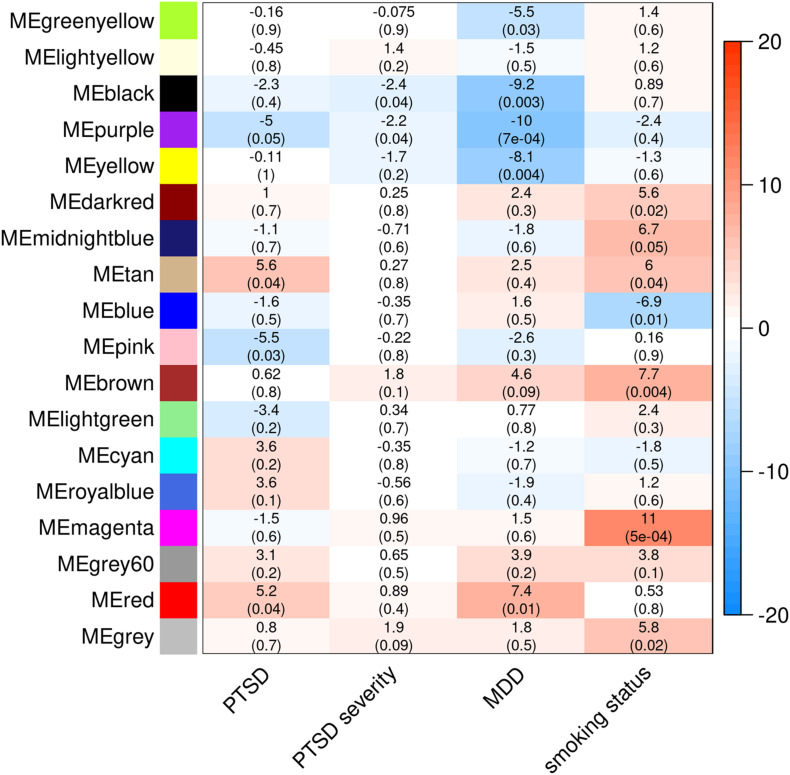
Co-expression modules detected in NHW MIRECC/Duke subjects and subsequent associations with PTSD, PTSD severity, MDD, and smoking status. The heatmap color scale represents effect sizes of each association (betas are listed in each cell, followed by corresponding *p*-value in parenthesis).

Weighted gene co-expression analysis in all MIRECC/Duke subjects identified 17 modules, none of which were associated with PTSD. When stratifying by ancestry, 16 independent co-expression modules were identified in the NHB subset and 18 were identified in the NHW subset. While none of the module eigengenes were associated with PTSD in the NHB subset, four modules significantly associated with PTSD status in the NHW subset (*p* ≤ 0.05, Figure 2). Two modules (MEtan and MEred) were upregulated in PTSD cases and two modules (MEpink and MEpurple) were downregulated in PTSD cases. MEpurple was also associated with PTSD severity as measured by total DTS score (*p* = 0.0356), and with current MDD status (*p* = 0.0007), an association that surpassed adjustment for multiple testing (Bonferroni adjustment; 18 tests; *p* = 0.0028). Additionally, MEred was also associated with MDD status (*p* = 0.0118) and MEtan was also associated with current smoking status (*p* = 0.041). These associations were in the same direction as were those for PTSD: MEpurple genes were downregulated in MDD and associated with decreasing total DTS score, MEred genes were upregulated in MDD, and MEtan genes were upregulated in smokers. Pathways significantly enriched in the PTSD-associated co-expression modules are listed in Table 3. The genes in MEtan showed significant enrichment for gene ontology (GO) terms involving plasma membrane/transmembrane, glycoprotein, and signal peptide (FDR *q* < 0.05), whereas genes in MEred were enriched for protein transport, alternative splicing, and phosphoprotein GO terms (FDR *q* < 0.05). MEpink genes were enriched for KEGG pathway term “metabolic pathways” (FDR *q* < 0.05) and MEpurple showed enrichment for actin binding, alternative splicing, and adaptive immunity GO terms (FDR *q* < 0.05).

**TABLE 3:** Pathways enriched in co-expression modules that are also associated with PTSD (MEpurple, MEtan, MEpink, and MEred).

Module	Category	Term	*N*	%	*p*-value	Fold Enrichment	FDR *q*-value
MEpurple	GOTERM_CC_DIRECT	GO:0016020∼membrane	84	24.21	3.29E-05	1.51	0.0124
MEpurple	UP_KEYWORDS	Actin-binding	18	5.19	4.08E-05	3.08	0.0126
MEpurple	UP_KEYWORDS	ATP-binding	50	14.41	4.43E-05	1.78	0.0069
MEpurple	GOTERM_MF_DIRECT	GO:0003779∼actin binding	19	5.48	8.22E-05	2.80	0.0415
MEpurple	UP_KEYWORDS	Alternative splicing	236	68.01	6.20E-04	1.13	0.0472
MEpurple	UP_KEYWORDS	Disease mutation	66	19.02	6.91E-04	1.48	0.0422
MEpurple	UP_KEYWORDS	Adaptive immunity	13	3.75	8.78E-04	3.01	0.0447
MEtan	GOTERM_CC_DIRECT	GO:0005886∼plasma membrane	56	36.13	4.22E-06	1.77	0.0008
MEtan	UP_KEYWORDS	Membrane	84	54.19	5.78E-06	1.48	0.0013
MEtan	UP_KEYWORDS	Glycoprotein	47	30.32	4.32E-05	1.78	0.0049
MEtan	UP_KEYWORDS	Transmembrane	61	39.35	5.50E-05	1.58	0.0041
MEtan	UP_SEQ_FEATURE	glycosylation site:N-linked (GlcNAc…)	44	28.39	6.92E-05	1.80	0.0388
MEtan	UP_SEQ_FEATURE	signal peptide	37	23.87	8.02E-05	1.93	0.0227
MEtan	UP_KEYWORDS	Transmembrane helix	60	38.71	9.65E-05	1.56	0.0054
MEtan	UP_SEQ_FEATURE	Transmembrane region	55	35.48	1.31E-04	1.60	0.0247
MEtan	GOTERM_CC_DIRECT	GO:0005887∼integral component of plasma membrane	24	15.48	3.69E-04	2.20	0.0339
MEtan	GOTERM_CC_DIRECT	GO:0016021∼integral component of membrane	56	36.13	5.86E-04	1.50	0.0359
MEtan	UP_KEYWORDS	Lipoprotein	18	11.61	6.28E-04	2.51	0.0280
MEpink	KEGG_PATHWAY	hsa01100:Metabolic pathways	30	14.78	1.72E-04	1.93	0.0274
MEred	UP_KEYWORDS	Protein transport	70	7.43	9.68E-06	1.62	0.0037
MEred	UP_KEYWORDS	Alternative splicing	619	65.71	1.43E-04	1.08	0.0271
MEred	UP_KEYWORDS	Phosphoprotein	556	59.02	1.94E-04	1.09	0.0245
MEred	UP_KEYWORDS	Ubl conjugation pathway	67	7.11	4.55E-04	1.47	0.0426

*Functional annotation source (Category), the enriched term associated with the module gene list (term), number of genes involved in the functional annotation term (N), percentage of involved genes to total genes (%), *p*-value (*p*-value), fold enrichment (Fold enrichment), and Benjamini-Hochberg false discovery rate (FDR *q*-value) are listed above.*

## Discussion

This is the largest meta-analysis of differential PTSD gene expression to date. Results not only identified genes in pathways relevant to PTSD pathogenesis, but also showed PTSD and MDD GWAS signals may be driven by differences in gene expression. The most significant differentially expressed gene was interleukin-1ß (*IL1B*), a pro-inflammatory cytokine that is associated with innate immunity and has been extensively studied for its role in autoinflammatory diseases (Dinarello, 2011). Indeed, those with PTSD are at increased risk for autoimmune disorders including rheumatoid arthritis, systemic lupus erythematosus, inflammatory bowel diseases, and multiple sclerosis (Ali et al., 2014; O’Donovan et al., 2015; Song et al., 2018; Bookwalter et al., 2020), and display elevated levels of pro-inflammatory cytokines, including interleukin-1ß (Spivak et al., 1997; Tursich et al., 2014; Passos et al., 2015). Moreover, inflammation and dysregulation of the immune system has been associated with other psychiatric disorders that frequently co-occur with PTSD such as MDD (Howren et al., 2009; Dowlati et al., 2010), schizophrenia (Miller et al., 2011), and alcohol use disorder (Crews et al., 2017; Patel et al., 2019). Animal studies have shown that mice subjected to a model of depression display increased levels of interleukin-1ß in the hippocampus (Goshen et al., 2008), a brain region integral to the neuropathology of PTSD due to its role in fear learning and contextual processing (Maren et al., 2013), as well as memory formation and retrieval (Bremner et al., 1997); in fact, a recent study in post-mortem human brain observed increased *IL1B* expression in dlPFC of PTSD cases compared to controls (Girgenti et al., 2021). The increase in *IL1B* expression observed among PTSD cases in the present research adds additional evidence to the growing body of work demonstrating widespread inflammatory response and dysregulation of the immune system in PTSD pathology.

We also detected reduced expression of *ILK*, the gene encoding ILK, a key scaffold protein that localizes to focal adhesions and is involved in the regulation of many cellular processes including cell growth, survival, adhesion, invasion, and migration. *ILK* is highly expressed in several brain regions including hippocampus, cerebellum, and frontal cortex (Mills et al., 2003; Xu et al., 2015) and is essential for neurite outgrowth (Mills et al., 2003), dendritogenesis (Naska et al., 2006), and survival signaling in hippocampal neurons (Gary et al., 2003). Although this gene has not previously been implicated in PTSD, indirect evidence points to *ILK* as a plausible candidate for further interrogation. In mouse, knockdown of brain-derived neurotrophic factor (BDNF), a gene associated not only with PTSD, but also MDD, bipolar disorder, and schizophrenia, results in dysregulation of hippocampal neurogenesis and memory deficits (BDNF+/-; Ernfors et al., 1994). Overexpression of ILK in the hippocampus of these mice rescues the hippocampal neurogenesis and memory deficits (Xu et al., 2015). Additionally, in a rat model of fetal alcohol spectrum disorder, alcohol-exposed pups displayed impaired contextual fear conditioning and memory performance, along with reduced hippocampal ILK activity (Bhattacharya et al., 2015). This pattern has also been observed in a mouse model for Alzheimer’s disease (AD), where ILK protein levels are significantly decreased in the hippocampus of APP/PS1 mice, which display perturbed neurogenesis and memory deficits, and can be rescued by overexpressing ILK (Xu et al., 2018). The direction of the effects in these animal studies is consistent with our observation that *ILK* expression is reduced in PTSD cases. Relatedly, several studies have reported a bi-directional association between PTSD and dementia (reviewed in Desmarais et al., 2020); those with PTSD are at increased risk for dementia and vice versa. More research is necessary to investigate the potential therapeutic value of ILK signaling pathway targets in PTSD, as have been suggested for AD (Li et al., 2012; Xu et al., 2018).

In addition to *IL1B* and *ILK*, pathway analysis of all gene expression probes nominally associated with PTSD revealed an enrichment of toll-like receptor (TLR) and interleukin-1 receptor (IL-1R) genes that share the conserved toll/IL-1R homologous region (TIR) and are involved in innate antibacterial and antifungal immunity, consistent with previous reports from gene expression (Breen et al., 2015) and methylation studies (Uddin et al., 2010; Smith et al., 2011) in PTSD. TLRs play an important role in pathogen recognition and activate a signaling cascade which leads to upregulation of pro-inflammatory cytokines, including interleukin-1ß. Identification of not only *IL1B*, but also several TLR genes, demonstrates an increased burden of dysregulated innate immunity and inflammatory response genes among PTSD cases.

Correlation network analysis identified many modules of co-expression in the MIRECC/Duke cohort. Four modules identified in the NHW samples were associated with PTSD, two of which were also associated with MDD. Identification of coordinated expression associated with both PTSD and MDD confirms not only a strong correlation between the two phenotypes, but also shared biological pathways underlying the two conditions. We also identified eQTLs that were only significant in those with either PTSD or MDD, demonstrating genotype × environment interactions affecting gene expression. For instance, rs28842268 was associated with adenylate kinase 4 (*AK4*) expression, but only among PTSD cases; there was no effect among the control samples. AK4 localizes to the mitochondrial matrix and is involved in the cell survival response to oxidative stress (Liu et al., 2009; Kong et al., 2013), a molecular state that is activated by the persistent hyperarousal and fear experienced by those with PTSD (reviewed in Miller et al., 2018). Using a convergent functional genomics approach, Le-Niculescu et al. (2009) implicated *AK4* in bipolar disorder, another neuropsychiatric condition which frequently co-occurs with PTSD (Cerimele et al., 2017). These data suggest there are genetic regulators of expression that are specific to the elevated stress and inflammatory state induced by PTSD.

Utilizing summary statistics from PTSD and MDD GWASs (Wray et al., 2018; Maihofer et al., submitted), we identified four genes (*UBA7*, *HLA-F*, *HSPA1B*, and *RERE*) with high probability of a shared causal eQTL variant with PTSD and/or MDD GWAS variants, thereby providing a potential mechanism by which the GWAS variant contributes to disease risk. *UBA7* encodes an E1 ubiquitin-activating enzyme shown to be involved in STAT1-mediated interferon-α (INF-α) regulation of hippocampal neurogenesis and apoptosis (Borsini et al., 2018), processes triggered by a pro-inflammatory state. The *UBA7* eQTL shares genetic effects with both PTSD and MDD GWAS variants, suggesting a potential pleiotropic mechanism at this locus. Recently, *UBA7* was implicated in a transcriptome-wide association study (TWAS) of PTSD using brain tissue (Girgenti et al., 2021), confirming the association we observe in whole blood. The summary statistics utilized in Girgenti et al. were obtained from MVP (Stein et al., 2021) as opposed to the current study, which used summary statistics from PGC-PTSD (Maihofer et al., submitted), demonstrating an independent replication of the *UBA7* finding. We also identified two eQTLs involving major histocompatibility complex (MHC) genes, *HLA-F* and *HSPA1B*, that displayed evidence for a shared genetic effect with MDD GWAS variants. The MHC group of genes encode proteins integral to immune response and variants in MHC genes have been associated with increased risk for many autoimmune diseases (reviewed in Matzaraki et al., 2017). As mentioned above, those with PTSD are at increased risk for autoimmune disorders and display increased levels of pro-inflammatory cytokines, including interleukin-1ß. The identification of multiple shared genetic effects of MHC gene expression and MDD risk increases the body of evidence implicating inflammatory and immune response pathways in both PTSD and MDD. Finally, two separate eQTLs for Arginine-Glutamic Acid Dipeptide Repeats (*RERE*) colocalize with MDD GWAS variants. Of note, a chromatin accessibility QTL (cQTL) in *RERE* identified in dlPFC of post-mortem human brains was shown to colocalize with schizophrenia GWAS variants (Bryois et al., 2018), another psychiatric disorder that co-occurs with PTSD.

The majority of these findings represent eQTLs from a PTSD-enriched dataset that colocalize with MDD GWAS variants. This further demonstrates not only the phenotypic comorbidity between PTSD and MDD, but identifies genomic regions of possible pleiotropy. Functional studies are necessary to further investigate these associations. Still, these results suggest biological mechanisms by which non-coding and common genetic variants may influence risk for PTSD and/or MDD.

While we have identified plausible novel candidates for PTSD, and confirmed many previously reported genes and pathways, this study is not without limitations. We acknowledge that the associations we observed in the differential expression analysis failed to achieve correction for multiple testing. While we have assembled the largest study of gene expression in PTSD to date, still larger sample sizes are required to reach an adequate power threshold to detect more modest differences. Despite this, *IL1B* has been repeatedly implicated in PTSD, therefore our replication of increased *IL1B* expression in PTSD cases is important. Because *ILK* has not previously been associated with PTSD, replication in independent datasets is necessary. Secondly, gene expression was measured in peripheral blood as opposed to brain tissue. We did attempt to impute genetically-regulated dlPFC expression in the NHW MIRECC/Duke subjects using a large eQTL reference panel (Huckins et al., 2019), but were only able to impute 6,928 genes with high confidence. Among the genes that were differentially expressed in both blood and imputed brain, nearly 70% showed a concordant direction of association with PTSD. The high percentage of concordance, as well as replication of pathways from the few brain-based studies available, lends credence to the hypothesis of at least partial overlap between blood and brain transcriptomes. Third, despite having compiled a large veteran cohort with respect to gene expression, it is difficult to identify robust interactions, particularly when SNPs involved have low minor allele frequency (MAF). As such, the eQTL interactions we have identified, particularly those involving SNPs with lower MAF, should be viewed as preliminary and need to be replicated in larger studies. Also, we did not investigate sex-specific associations due to the relatively small number of female samples available in the current study. Ascertainment of female veterans should be prioritized to facilitate research of possible sex-specific genetic factors influencing PTSD risk. Finally, gene expression in this study was determined using microarrays and not RNA-sequencing. Future studies that interrogate gene expression using sequencing approaches would be beneficial to obtain a truly global depiction of the transcriptome.

## Conclusion

This is the largest study of differential gene expression between PTSD cases and controls to date, and these analyses have identified many logical candidate genes and pathways for further research. Notably, while we did not replicate the *FKBP5* finding (*p* > 0.05), the present findings provide additional support for the role of *IL1B* and other inflammatory response and innate immunity pathway genes in PTSD, and have identified *ILK* as a novel candidate. Further, we identified modules of co-expressed genes that were associated with both PTSD and MDD, suggesting common biological pathways underlie the two comorbid disorders. Finally, we identified changes in gene expression that may help explain several PTSD and MDD GWAS signals. These data provide further insight into genetic factors and pathways associated with PTSD, as well as potential regions of pleiotropy between PTSD and MDD.

## Members of the VA Mid-Atlantic Mental Illness Research, Education, and Clinical Center (MIRECC) Workgroup

Mira Brancu, Ph.D., Patrick S. Calhoun, Ph.D., Eric Dedert, Ph.D., Eric B. Elbogen, Ph.D., John A. Fairbank, Ph.D., Robin A. Hurley, M.D., Jason D. Kilts, Ph.D., Angela Kirby, M.S., Sara Martindale, Ph.D., Scott D. McDonald, Ph.D., Scott D. Moore, M.D., Ph.D., Rajendra A. Morey, M.D., M.S., Jennifer C. Naylor, Ph.D., Jared Rowland, Ph.D., Robert Shura, Ph.D., Cindy Swinkels, Ph.D., Steven T. Szabo, M.D., Ph.D., Katherine H. Taber, Ph.D., Larry A. Tupler, Ph.D., and Ruth E. Yoash-Gantz, Psy.D.

## Members of the PTSD Initiative

Sarah McLeay, BSc(Hons), Ph.D., Wendy Harvey, BSc(Hons), MBBS, MPH, Madeline Romaniuk, BA, GradDipPsych, BBehSc(Hons), DPsych(Clinical), Darrell Crawford, MBBS, FRACP, M.D., David Colquhoun, MBBS, FRACP, Ross McD Young, Ph.D., Miriam Dwyer, BSc, HDipEd, John Gibson, MBBS, FRANZCP, Robyn O’Sullivan, MBBS, FRACP, Graham Cooksley, MBBS, M.D., FRACP, Christopher Strakosch, M.D., FRACP, Rachel Thomson, MBBS, GradDipClinEpi, Ph.D., FRACP, Joanne Voisey, BSc(Hons), Ph.D., Bruce Lawford, MBBS, FRANZCP, FAChAM (RACP).

## Data Availability Statement

The datasets presented in this article are not readily available because the institutional review board (IRB) protocols governing these datasets do not allow sharing of individual level data. Requests to access the datasets should be directed to the senior author, AA-K at allison.ashleykoch@duke.edu.

## Ethics Statement

The studies involving human participants were reviewed and approved by the Durham (NC) VA Medical Center IRB, McGuire (Richmond, VA) VA Medical Center IRB, Hampton (VA) VA Medical Center IRB, W.G. (Bill) Hefner (Salisbury, NC) VA Medical Center IRB, Greenslopes Research and Ethics Committee, Queensland University of Technology Human Research Committee, San Diego (CA) VA Medical Center IRB, Togus (ME) VA Medical Center IRB, Cincinnati (OH) VA Medical Center IRB, Puget Sound (WA) VA Medical Center IRB, Dartmouth College (NH) IRB, University of California San Diego (CA) IRB, and Human Research Protections Office of the Department of Defense. The patients/participants provided their written informed consent to participate in this study.

## Author Contributions

AA-K, MH, and MG contributed to the design of the study. DM, MD, CM, GG, MS, NK, JB, MH, and AA-K assisted in the data collection, generation, and preparation. MG, XQ, and DM performed the analyses. MG prepared the first draft of the manuscript. AA-K, MH, NK, and JB contributed to the comments and edits during manuscript drafting. All authors reviewed the manuscript, provided feedback, and approved the manuscript for submission.

## Conflict of Interest

The authors declare that the research was conducted in the absence of any commercial or financial relationships that could be construed as a potential conflict of interest.

## Publisher’s Note

All claims expressed in this article are solely those of the authors and do not necessarily represent those of their affiliated organizations, or those of the publisher, the editors and the reviewers. Any product that may be evaluated in this article, or claim that may be made by its manufacturer, is not guaranteed or endorsed by the publisher.

## Footnotes

1
https://www.mentalhealth.va.gov/docs/data-sheets/2020/2020-National-Veteran-Suicide-Prevention-Annual-Report-11-2020-508.pdf
