# Supplementary material for: Gene Expression Analysis in Three Posttraumatic Stress Disorder Cohorts Implicates Inflammation and Innate Immunity Pathways and Uncovers Shared Genetic Risk With Major Depressive Disorder
Source: Front Neurosci. 2021 Jul 29;15:678548. doi: 10.3389/fnins.2021.678548 (PMC8358297; doi:10.3389/fnins.2021.678548)
Supplement: Supplementary file 1 [file Data_Sheet_1.PDF]

Supplementary Material

1 Supplementary Figures and Tables

1.1 Supplementary Figures

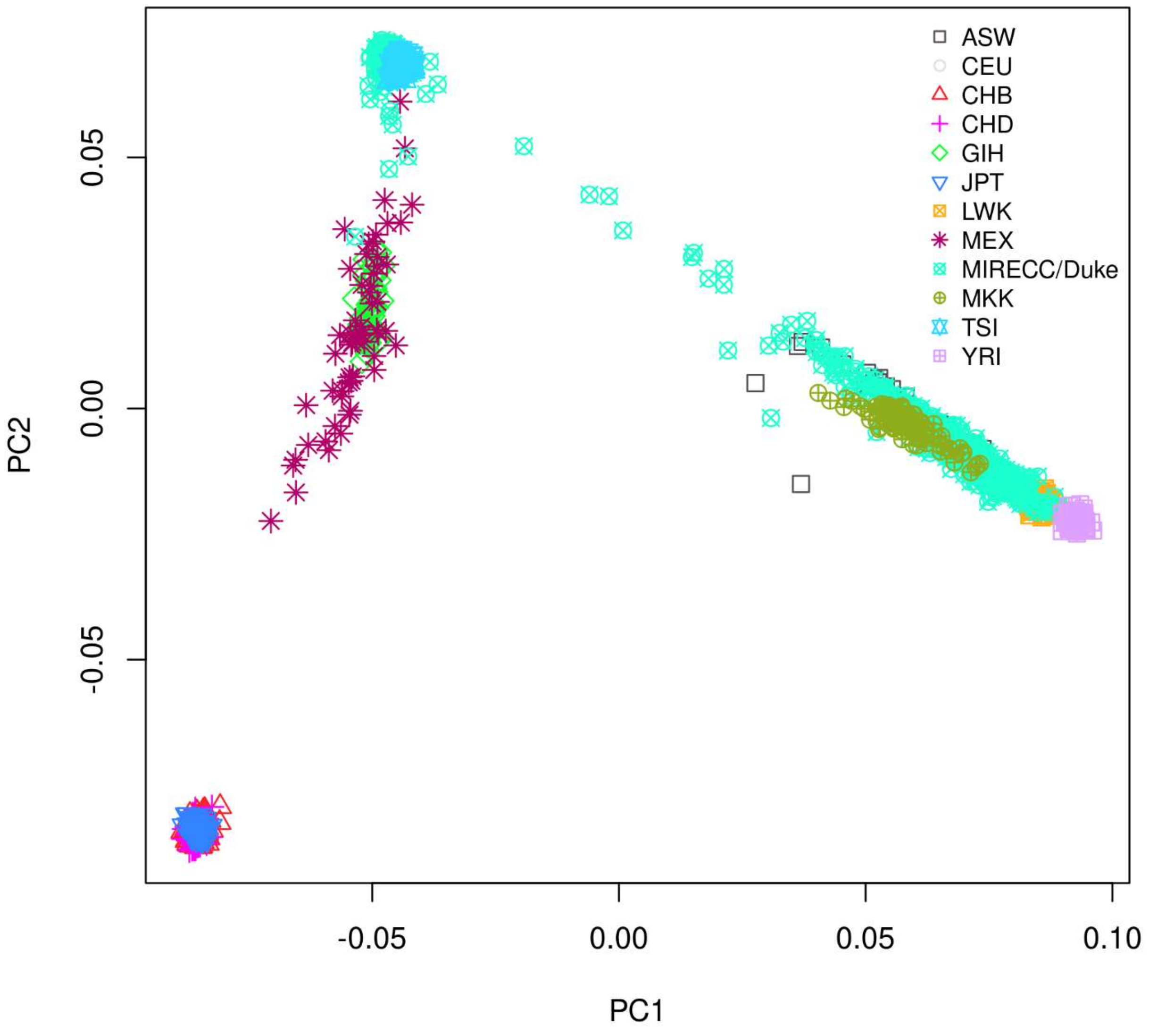

Supplementary Figure 1. PCA plot of MIRECC/Duke dataset overlaid on HapMap3.

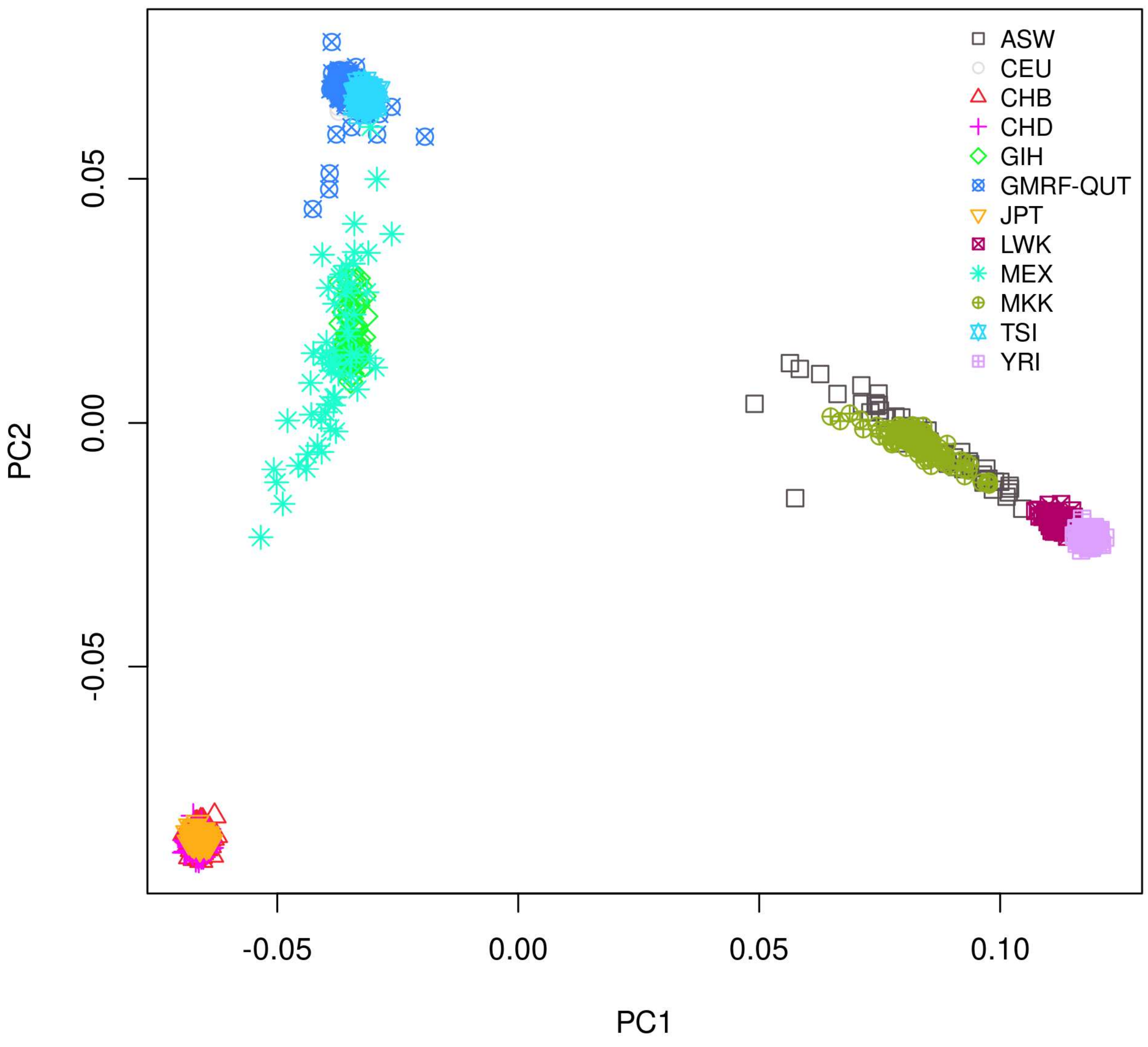

Supplementary Figure 2. PCA plot of GMRF-QUT dataset overlaid on HapMap3.

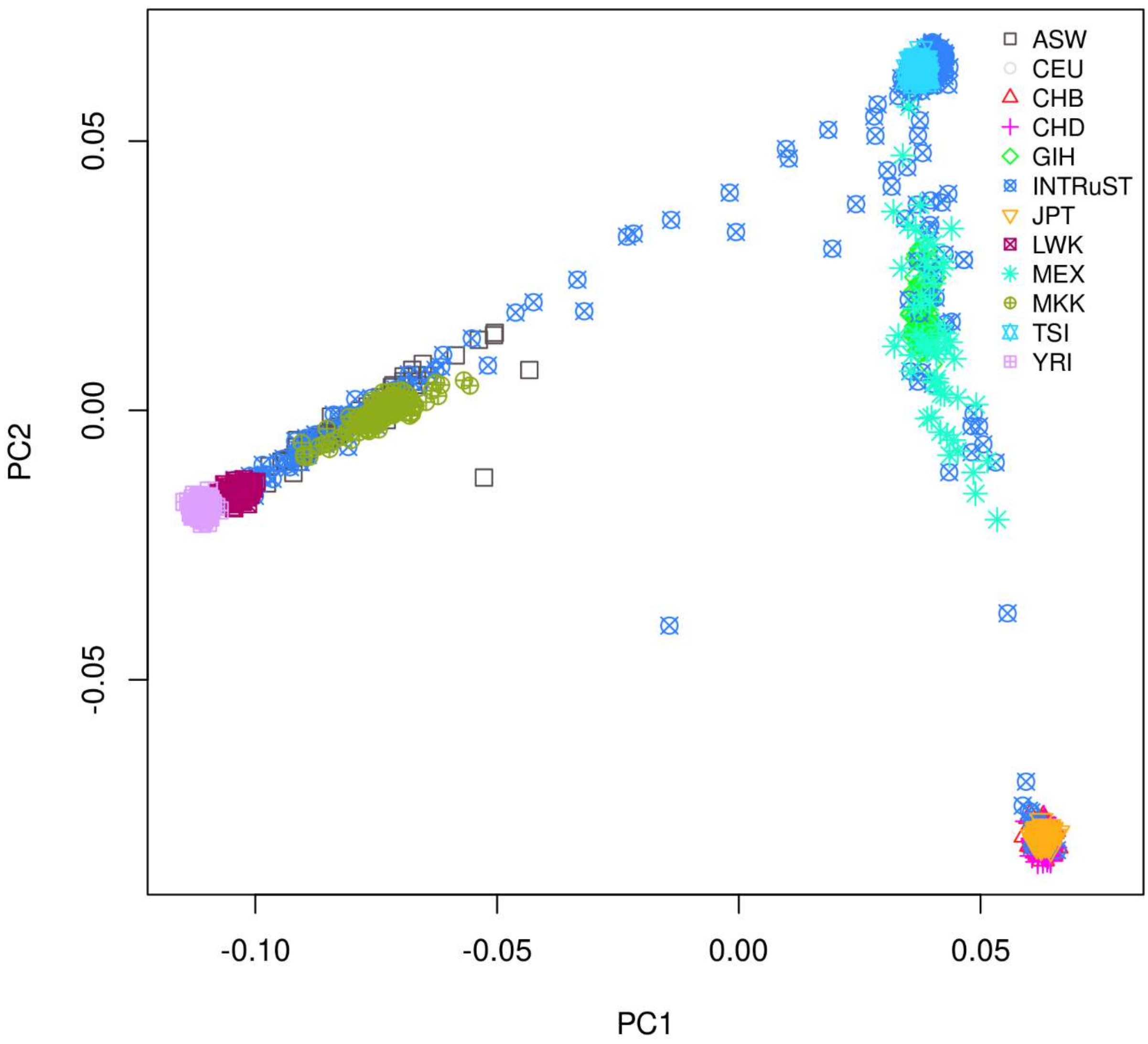

Supplementary Figure 3. PCA plot of INTRuST dataset overlaid on HapMap3.

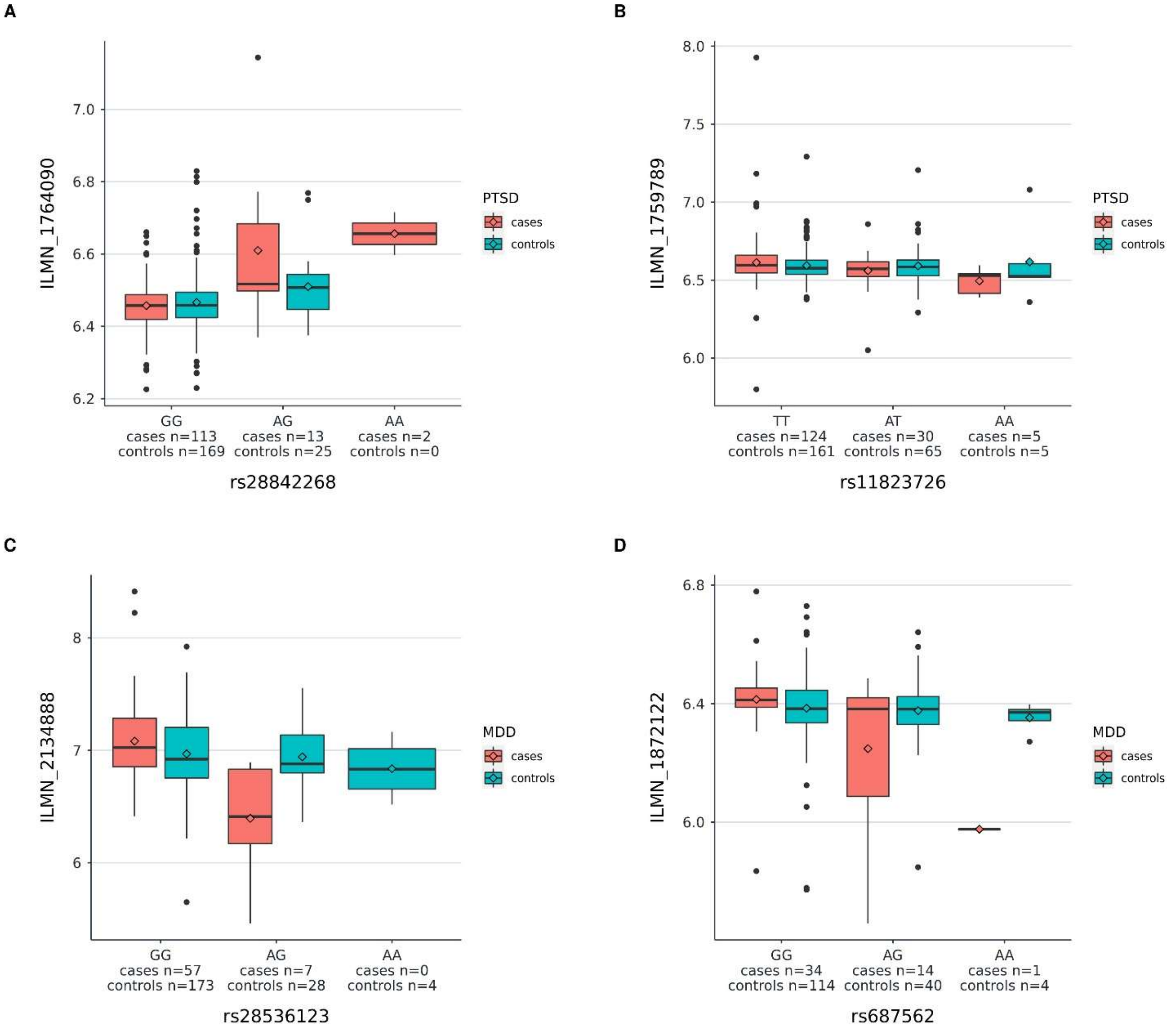

Supplementary Figure 4. eQTL interactions from an additive genetic model in the MIRECC/Duke cohort displayed as boxplots of the interquartile range (IQR) with all data points included for ease of interpretation: **(A)** rs28842268 by PTSD was associated with ILMN_1764090 in NHB subset **(B)** rs11823726 by PTSD was associated with ILMN_1759789 in NHB subset **(C)** rs28536123 by MDD was associated with ILMN_2134888 in NHW subset **(D)** rs687562 by MDD was associated with ILMN_1872122 in NHW subset. Genotypes are shown on the x-axis and gene expression is depicted on the y-axis. The red boxes represent cases (either PTSD or MDD) and the blue boxes represent control subjects. Diamonds in each box represent the mean gene expression value; horizonal lines in the boxes represent the median gene expression value. Samples sizes (n) for cases and controls are listed below each genotype.

1.2 Supplementary Tables

Table S1. Differential PTSD gene expression meta-analysis results (meta p-value, FDR q-value), including cohort-specific sample sizes (N), log fold change (logFC), and p-values.

				MIRECC/Duke			INTRuST			GMRF-QUT		
gene	probe	meta p-value	FDR q-value	N	logFC	p-value	N	logFC	p-value	N	logFC	p-value
IL1B	ILMN_1775501	2.15E-05	0.2474	718	0.1337	0.0002	151	0.2398	0.0095	96	0.0319	0.6103
ILK	ILMN_2364376	0.0001	0.6303	718	-0.0762	0.0001	152	-0.1171	0.0614	96	0.0224	0.6497
EXOC6	ILMN_1651628	0.0003	0.9367	718	0.1064	0.0001	151	0.0762	0.2792	96	-0.0074	0.8935
SLC37A3	ILMN_2307598	0.0005	0.9367	704	0.1077	0.0004	96	0.0586	0.3236	96	0.0205	0.6200
GK	ILMN_1725471	0.0005	0.9367	718	0.0985	0.0033	150	0.1447	0.0197	96	0.0355	0.5822
TMEM41B	ILMN_1678004	0.0006	0.9367	718	0.1002	6.13E-06	152	0.0143	0.8761	96	-0.0581	0.2060
HDAC3	ILMN_1772455	0.0006	0.9367	718	-0.0558	0.0016	152	-0.0524	0.2263	96	-0.0498	0.2537
ABI3	ILMN_1755658	0.0007	0.9367	718	-0.0773	0.0174	151	-0.2157	0.0005	96	-0.0220	0.7084
ACP2	ILMN_2104830	0.0008	0.9367	698	-0.0362	0.0095	121	-0.0665	0.0581	96	-0.0892	0.0936
LMNB1	ILMN_2126706	0.0012	0.9367	713	0.0917	0.0038	139	0.1437	0.0717	96	0.0381	0.5565
ANXA3	ILMN_1694548	0.0013	0.9367	714	0.1466	0.0221	144	0.2276	0.0619	96	0.1669	0.0418
GNA12	ILMN_2216157	0.0014	0.9367	704	0.0688	0.0154	146	0.1785	0.0273	96	0.0791	0.2269
INO80D	ILMN_3240685	0.0016	0.9367	711	0.1011	0.0008	132	0.0139	0.7645	96	0.0382	0.4205
ATP11B	ILMN_1658884	0.0016	0.9367	709	0.0775	0.0005	109	0.0310	0.3513	96	-0.0145	0.7282
TLR6	ILMN_1654560	0.0018	0.9367	718	0.0967	0.0027	152	0.1708	0.0453	96	-0.0207	0.7015
SNORA25	ILMN_1682038	0.0019	0.9367	718	0.0891	0.0010	151	0.1123	0.1411	96	-0.0230	0.5935
LOC399804	ILMN_3284119	0.0021	0.9367	718	0.1290	0.0078	139	0.2750	0.0065	96	-0.0208	0.6697
RAB8A	ILMN_1760858	0.0022	0.9367	718	-0.0396	0.0140	152	-0.0845	0.2682	96	-0.0939	0.0425
LOC645251	ILMN_3199647	0.0022	0.9367	695	0.0457	0.0141	134	0.0918	0.0412	96	0.0445	0.3696
ALDH6A1	ILMN_1785284	0.0023	0.9367	683	-0.0435	0.0060	117	-0.0422	0.2115	96	-0.0438	0.3148
FCGR1C	ILMN_3247506	0.0024	0.9367	715	0.1049	0.0105	143	0.1207	0.0730	96	0.0682	0.4145
SLC2A6	ILMN_1778321	0.0024	0.9367	718	-0.0926	0.0031	151	-0.1146	0.0998	96	0.0064	0.9069
PCCB	ILMN_1761010	0.0024	0.9367	718	-0.0736	0.0082	151	-0.0775	0.0643	96	-0.0190	0.6301
GOPC	ILMN_2388585	0.0026	0.9367	717	0.0633	0.0067	128	0.0843	0.0331	96	-0.0001	0.9979
NACAP1	ILMN_2145250	0.0028	0.9367	715	0.1041	0.0590	124	0.1534	0.0430	96	0.0984	0.0226
HMOX1	ILMN_1800512	0.0028	0.9367	717	-0.1269	0.0003	144	0.0018	0.9712	96	-0.0017	0.9800
WDR68	ILMN_1706706	0.0029	0.9367	715	-0.0690	0.0013	148	-0.0334	0.3997	96	0.0004	0.9918
ELP3	ILMN_1744068	0.0030	0.9367	718	-0.0507	0.0173	152	-0.1379	0.0070	96	0.0026	0.9431
GALM	ILMN_1671482	0.0030	0.9367	706	-0.0643	0.0017	142	-0.0217	0.5901	96	-0.0304	0.5746
TLR5	ILMN_1722981	0.0030	0.9367	718	0.1233	0.0003	152	-0.0810	0.4101	96	0.0568	0.3258
TADA3	ILMN_1674866	0.0031	0.9367	718	-0.0562	0.0149	152	-0.1302	0.0298	96	-0.0175	0.6939
VPS33A	ILMN_1662316	0.0031	0.9367	715	-0.0399	0.0016	136	-0.0076	0.8086	96	-0.0340	0.4402
DHRS4	ILMN_2185884	0.0032	0.9367	718	-0.0563	0.0220	151	-0.0911	0.1454	96	-0.0916	0.0986
TRIOBP	ILMN_1735788	0.0034	0.9367	718	-0.0628	0.0089	152	-0.0804	0.2614	96	-0.0625	0.2607
NAAA	ILMN_2285568	0.0036	0.9367	716	-0.1528	0.0001	136	0.0891	0.1846	96	-0.0055	0.9253

TUFM	ILMN_1738369	0.0036	0.9367	718	-0.0568	0.0237	152	-0.1141	0.0581	96	-0.0603	0.2914
GPR114	ILMN_1666902	0.0037	0.9367	707	-0.0814	0.0131	138	-0.1229	0.0205	96	0.0023	0.9728
UBA3	ILMN_2324157	0.0038	0.9367	713	0.0588	0.0069	130	0.0674	0.2137	96	0.0274	0.5197
ZDHHC17	ILMN_1697153	0.0038	0.9367	715	0.0967	0.0074	146	0.1427	0.1188	96	0.0148	0.7652
ABLIM1	ILMN_2396672	0.0040	0.9367	674	0.0955	0.0006	88	0.0535	0.2190	96	-0.0609	0.2072
SESN1	ILMN_1800626	0.0041	0.9367	713	0.0888	0.0035	139	0.0538	0.3967	96	0.0222	0.6665
LOC647307	ILMN_3285785	0.0041	0.9367	674	-0.1072	0.0091	105	-0.0629	0.2647	96	-0.0973	0.3353
RABGEF1	ILMN_2230577	0.0042	0.9367	673	0.0471	0.0002	123	-0.0106	0.6897	96	-0.0159	0.6524
GNL3	ILMN_2324056	0.0044	0.9367	711	0.0664	0.0073	130	0.0678	0.0706	96	-0.0055	0.9051
PHF20	ILMN_1813657	0.0047	0.9367	715	-0.0327	0.0315	152	-0.1371	0.0180	96	-0.0270	0.6136
RYK	ILMN_1658526	0.0047	0.9367	697	0.0436	0.0040	111	0.0331	0.2484	96	0.0020	0.9547
GK	ILMN_2393296	0.0048	0.9367	718	0.0912	0.0069	150	0.0724	0.3198	96	0.0440	0.4818
LOC643402	ILMN_1705749	0.0050	0.9367	666	-0.1039	0.0288	117	-0.0336	0.6199	96	-0.1137	0.0104
FTSJ1	ILMN_2401822	0.0052	0.9367	714	-0.0345	0.1225	129	-0.0658	0.0618	96	-0.1198	0.0060
IL18RAP	ILMN_1721762	0.0054	0.9367	718	0.1609	0.0001	152	-0.1413	0.2801	96	0.0093	0.9395
PROK2	ILMN_1775257	0.0054	0.9367	718	0.0921	0.0166	152	0.2636	0.0369	96	0.0041	0.9705
EXOC1	ILMN_2347805	0.0058	0.9367	712	0.0757	0.0056	136	0.0912	0.0521	96	-0.0371	0.4134
SNX13	ILMN_1797082	0.0060	0.9367	700	0.0601	0.0088	119	0.0592	0.1316	96	0.0041	0.9185
ARRB1	ILMN_2325168	0.0061	0.9367	718	-0.0681	0.0052	149	-0.0471	0.4780	96	-0.0305	0.5926
YIF1B	ILMN_2363668	0.0061	0.9367	718	-0.0745	0.0024	152	-0.0444	0.4703	96	0.0064	0.9025
TNFRSF8	ILMN_1659257	0.0062	0.9367	711	-0.0457	0.0186	144	-0.0615	0.0679	96	-0.0236	0.7204
NCOR2	ILMN_2340052	0.0062	0.9367	718	-0.0527	0.1197	152	-0.1464	0.0344	96	-0.1147	0.0319
LOC728590	ILMN_3301065	0.0063	0.9367	718	0.0490	0.0449	152	0.1738	0.0925	96	0.1144	0.1458
	ILMN_1849013	0.0064	0.9367	717	0.1320	0.0011	146	0.0862	0.4562	96	-0.0464	0.4011
SMARCB1	ILMN_1758823	0.0064	0.9367	718	-0.0828	0.0012	152	0.0393	0.4865	96	-0.0453	0.2880
CD44	ILMN_1778625	0.0064	0.9367	708	0.0777	0.0173	140	0.0971	0.0811	96	0.0156	0.7228
NAMPT	ILMN_1653871	0.0065	0.9367	718	0.0769	0.0494	152	0.3549	0.0105	96	0.0398	0.6699
IVD	ILMN_1724207	0.0066	0.9367	668	-0.0308	0.0269	81	-0.0436	0.1241	96	-0.0598	0.2448
SSBP4	ILMN_2327346	0.0066	0.9367	713	-0.0519	0.0519	145	-0.1098	0.0258	96	-0.0534	0.3698
GPN2	ILMN_2205245	0.0067	0.9367	717	-0.0437	0.0225	148	-0.0860	0.0547	96	-0.0151	0.7387
CDCA4	ILMN_1684045	0.0067	0.9367	718	-0.0397	0.0134	126	-0.0242	0.3005	96	-0.0415	0.3626
C17orf58	ILMN_2398926	0.0068	0.9367	713	-0.0440	0.0069	126	-0.0089	0.7226	96	-0.0404	0.2982
PIN1	ILMN_1776375	0.0069	0.9367	718	-0.0310	0.0760	152	-0.1255	0.0145	96	-0.0422	0.3085
CAPN2	ILMN_1716057	0.0070	0.9367	699	-0.0356	0.0141	120	-0.0384	0.2223	96	-0.0294	0.4830
ILVBL	ILMN_1769451	0.0071	0.9367	715	-0.0498	0.0237	140	-0.0907	0.0328	96	-0.0047	0.9212
CEPT1	ILMN_1676588	0.0071	0.9367	718	0.0784	0.0151	151	0.1560	0.0858	96	0.0055	0.9088
P2RY13	ILMN_1664094	0.0071	0.9367	718	0.0453	0.0445	152	0.1028	0.1060	96	0.0988	0.1642
ARMC6	ILMN_1697118	0.0072	0.9367	714	-0.0405	0.2067	142	-0.0624	0.2325	96	-0.2383	0.0001
GMPR2	ILMN_2365595	0.0072	0.9367	718	0.0370	0.0155	152	0.1217	0.0596	96	-0.0039	0.9258
HNRNPH2	ILMN_2362245	0.0073	0.9367	713	0.0806	0.0279	133	0.1517	0.0468	96	0.0259	0.6598
PLIN2	ILMN_2138765	0.0076	0.9367	718	-0.0566	0.0082	150	-0.0346	0.5137	96	-0.0416	0.4321
LOC100128460	ILMN_3187283	0.0077	0.9367	704	0.1132	0.0215	127	0.1591	0.3586	96	0.1024	0.1704

CD300C	ILMN_1701906	0.0077	0.9367	702	-0.0859	0.0066	133	-0.0884	0.1148	96	0.0366	0.5926
C7orf50	ILMN_1718336	0.0078	0.9367	718	-0.0655	0.0088	152	-0.0523	0.4476	96	-0.0402	0.4876
LOC100132291	ILMN_3275345	0.0078	0.9367	718	0.0574	0.0201	152	0.1701	0.1687	96	0.0440	0.4685
MID1IP1	ILMN_2165473	0.0079	0.9367	718	-0.1134	0.0014	151	-0.0021	0.9750	96	-0.0013	0.9818
MED4	ILMN_1664641	0.0079	0.9367	718	0.1046	0.0007	150	0.0266	0.7247	96	-0.0561	0.3641
FAM13AOS	ILMN_3245879	0.0080	0.9367	707	0.0458	0.0351	136	0.0850	0.0120	96	-0.0020	0.9659
CSF1R	ILMN_1686623	0.0080	0.9367	718	-0.0605	0.0736	152	-0.2247	0.0261	96	-0.0860	0.2776
LRFN3	ILMN_2103919	0.0081	0.9367	715	-0.0622	0.0129	134	-0.0654	0.1960	96	-0.0185	0.7137
PHTF1	ILMN_1803464	0.0081	0.9367	706	0.0552	0.0133	134	0.0447	0.2329	96	0.0220	0.6052
SLFN13	ILMN_2215862	0.0081	0.9367	705	-0.0465	0.0414	133	-0.0742	0.0729	96	-0.0375	0.3241
LOC646819	ILMN_3287093	0.0081	0.9367	718	0.0619	0.0140	152	0.1466	0.1504	96	0.0217	0.8138
HCFC1R1	ILMN_1757877	0.0082	0.9367	718	-0.0477	0.0871	152	-0.1697	0.0099	96	-0.0445	0.4085
CCDC117	ILMN_1809889	0.0083	0.9367	718	0.0720	0.0074	143	0.1003	0.1375	96	-0.0230	0.6624
FCGR1B	ILMN_2261600	0.0083	0.9367	690	0.0877	0.0183	147	0.1235	0.1956	96	0.0656	0.5071
PPM1G	ILMN_1806867	0.0084	0.9367	718	-0.0513	0.0220	152	-0.1145	0.1009	96	-0.0153	0.6914
LOC644739	ILMN_1660320	0.0085	0.9367	420	0.0331	0.0345	147	0.0928	0.2591	96	0.0877	0.1612
MFSD5	ILMN_1702065	0.0086	0.9367	716	-0.0502	0.0143	151	-0.0956	0.0963	96	0.0038	0.9352
C20orf100	ILMN_2082209	0.0086	0.9367	717	-0.0500	0.0637	149	-0.0903	0.0290	96	-0.0456	0.3681
GMPR2	ILMN_1677919	0.0087	0.9367	718	0.0669	0.0075	152	0.0798	0.1086	96	-0.0273	0.5215
USP24	ILMN_1684594	0.0087	0.9367	718	-0.0343	0.1299	152	-0.0788	0.1394	96	-0.1440	0.0073
	ILMN_1896967	0.0087	0.9367	706	0.0617	0.0331	129	0.0946	0.1098	96	0.0541	0.3703
SNAP47	ILMN_3243982	0.0089	0.9367	703	-0.0468	0.0056	98	-0.0038	0.8666	96	-0.0271	0.5002
FAH	ILMN_1781536	0.0090	0.9367	707	-0.0524	0.0141	107	-0.0867	0.0661	96	0.0089	0.8352
STK17B	ILMN_1798543	0.0092	0.9367	718	0.0696	0.0256	150	0.2357	0.0108	96	-0.0306	0.4967
DPH5	ILMN_1770048	0.0093	0.9367	697	0.0871	0.0028	137	0.0312	0.5481	96	-0.0162	0.7502
KIAA0040	ILMN_1797191	0.0094	0.9367	715	0.0417	0.0271	136	0.0385	0.1778	96	0.0371	0.3801
PAPSS2	ILMN_2410929	0.0095	0.9367	608	-0.0293	0.0209	117	-0.0664	0.0550	96	0.0004	0.9911
DHRS4L2	ILMN_1761450	0.0095	0.9367	697	-0.0318	0.0248	115	-0.0390	0.1875	96	-0.0432	0.4069
FAM54B	ILMN_3264466	0.0097	0.9367	716	-0.0356	0.0229	141	-0.0286	0.3762	96	-0.0570	0.2240
CEBPA	ILMN_1715715	0.0100	0.9367	718	-0.0637	0.0244	152	-0.0969	0.1436	96	-0.0270	0.5888
SEC61A1	ILMN_1659564	0.0102	0.9367	718	-0.0324	0.1496	152	-0.1540	0.0225	96	-0.0907	0.0908
PDCD6IP	ILMN_1693259	0.0103	0.9367	718	0.0635	0.0287	147	0.1453	0.0123	96	-0.0299	0.5498
C7orf26	ILMN_1730048	0.0103	0.9367	718	-0.0500	0.0141	151	-0.0349	0.5781	96	-0.0464	0.2786
RPS15A	ILMN_2337241	0.0104	0.9367	718	0.0498	0.0154	152	0.0985	0.2345	96	0.0336	0.7169
C22orf30	ILMN_1741295	0.0105	0.9367	700	-0.1316	0.0605	147	0.0247	0.8674	96	-0.2450	0.0004
RBM27	ILMN_3248026	0.0106	0.9367	717	0.0641	0.0077	146	0.0129	0.7667	96	0.0364	0.4284
GIYD1	ILMN_2368575	0.0106	0.9367	717	-0.0552	0.0240	152	-0.0691	0.1484	96	-0.0234	0.6385
RCOR3	ILMN_1682095	0.0109	0.9367	718	0.0831	0.0112	152	0.0999	0.3344	96	0.0185	0.7753
SPI1	ILMN_2392043	0.0111	0.9367	718	-0.0892	0.0039	152	0.0335	0.7000	96	-0.0675	0.3138
GNG10	ILMN_1757074	0.0112	0.9367	716	0.1049	0.0384	149	0.3389	0.0467	96	0.0253	0.8020
MDH2	ILMN_2079004	0.0113	0.9367	718	-0.0539	0.0003	152	-0.0078	0.8656	96	0.0749	0.1081
CLK1	ILMN_1679727	0.0113	0.9367	718	0.1222	0.0076	149	0.2380	0.1866	96	-0.0402	0.5686
TCL1B	ILMN_2382309	0.0114	0.9367	709	0.0542	0.0222	142	0.0140	0.7604	96	0.0680	0.0862

KCNJ2	ILMN_1780334	0.0114	0.9367	718	0.0877	0.0149	152	0.0711	0.4834	96	0.0565	0.4009
FBXO38	ILMN_1754811	0.0115	0.9367	718	0.0655	0.0033	149	-0.0054	0.9303	96	0.0201	0.6706
ACTL6A	ILMN_2328280	0.0115	0.9367	714	0.0457	0.0505	134	0.0956	0.0442	96	0.0240	0.5731
LOC100128998	ILMN_3234254	0.0117	0.9367	422	-0.0609	0.1717	69	-0.0099	0.9417	96	-0.1917	0.0003
LOC648605	ILMN_1737574	0.0117	0.9367	715	-0.1255	0.0011	142	0.0168	0.6492	96	0.0037	0.9327
SYT11	ILMN_1717934	0.0118	0.9367	718	-0.0587	0.0420	152	-0.1502	0.0184	96	0.0097	0.8522
YTHDF3	ILMN_1657470	0.0119	0.9367	718	0.0626	0.0129	152	0.0590	0.4161	96	0.0245	0.6119
SNAP29	ILMN_1659857	0.0120	0.9367	715	-0.0369	0.0775	139	-0.0666	0.0508	96	-0.0585	0.2787
LOC88523	ILMN_1737561	0.0120	0.9367	718	0.0788	0.0112	152	0.0461	0.6596	96	0.0475	0.4092
ZC3H11B	ILMN_3303965	0.0121	0.9367	718	0.0513	0.0371	152	0.1574	0.0758	96	0.0184	0.7044
RHOC	ILMN_2313730	0.0121	0.9367	718	-0.0585	0.0548	152	-0.2187	0.0087	96	0.0159	0.8076
PSMD6	ILMN_1779633	0.0121	0.9367	715	0.0420	0.1156	147	0.1514	0.0152	96	0.0424	0.3284
ARRB1	ILMN_1730620	0.0122	0.9367	718	-0.0681	0.0037	152	-0.0657	0.2511	96	0.0480	0.2785
SPTAN1	ILMN_2095133	0.0122	0.9367	717	-0.0685	0.0941	148	-0.1473	0.0734	96	-0.0797	0.1404
CHD4	ILMN_1658411	0.0123	0.9367	718	-0.0505	0.1026	152	-0.1558	0.0923	96	-0.0773	0.0873
PTEN	ILMN_1701134	0.0123	0.9367	718	0.0575	0.0741	152	0.1894	0.0309	96	0.0318	0.4877
PTTG1	ILMN_2042771	0.0123	0.9367	718	-0.0597	0.0360	152	-0.1063	0.0622	96	-0.0123	0.8343
LUZP1	ILMN_1714159	0.0124	0.9367	706	-0.0337	0.1124	130	-0.0785	0.0266	96	-0.0559	0.2110
SLC9A9	ILMN_1758315	0.0124	0.9367	707	-0.0352	0.0214	87	0.0078	0.7728	96	-0.0787	0.0480
PRKCB1	ILMN_1716563	0.0124	0.9367	718	0.0681	0.0276	150	0.1346	0.0615	96	-0.0046	0.9290
LOC345041	ILMN_3283680	0.0125	0.9367	717	0.0758	0.0170	146	0.0862	0.2567	96	0.0160	0.7481
RER1	ILMN_1760933	0.0126	0.9367	715	-0.0442	0.0070	130	-0.0281	0.3187	96	0.0180	0.7192
DUSP5	ILMN_1656501	0.0128	0.9367	718	-0.0905	0.0015	148	0.0185	0.7229	96	0.0003	0.9954
NAMPT	ILMN_1753111	0.0129	0.9367	718	0.0950	0.0404	146	0.2810	0.0995	96	0.0601	0.5682
LOC645175	ILMN_3215381	0.0133	0.9367	694	0.0382	0.0115	124	0.0370	0.2137	96	-0.0100	0.8204
AGBL5	ILMN_2308689	0.0134	0.9367	696	-0.0383	0.0079	140	-0.0200	0.5262	96	-0.0056	0.9025
SULT1B1	ILMN_1733443	0.0136	0.9367	626	0.0441	0.0042	132	0.0255	0.4954	96	-0.0123	0.6726
FXR1	ILMN_1679640	0.0136	0.9367	717	0.0619	0.0197	150	0.0590	0.3662	96	0.0298	0.5114
TBC1D13	ILMN_1662161	0.0137	0.9367	713	-0.0323	0.0937	128	-0.0238	0.4148	96	-0.0945	0.0114
RNF121	ILMN_2356031	0.0137	0.9367	714	-0.0437	0.0292	144	-0.0590	0.1325	96	-0.0144	0.7448
SLC35E1	ILMN_1752333	0.0137	0.9367	716	-0.0416	0.0189	152	-0.0240	0.6670	96	-0.0554	0.2291
CLEC2B	ILMN_1784608	0.0138	0.9367	706	0.1022	0.0063	149	0.0590	0.5480	96	-0.0034	0.9546
LGALS8	ILMN_2353358	0.0138	0.9367	718	0.0574	0.0431	143	0.0800	0.1643	96	0.0432	0.3733
SHPK	ILMN_2216918	0.0138	0.9367	713	-0.0453	0.0294	144	-0.0635	0.0866	96	-0.0035	0.9512
CNDP2	ILMN_1726769	0.0142	0.9367	718	-0.0399	0.1062	152	-0.0991	0.1420	96	-0.0749	0.0639
PGAM5	ILMN_1788254	0.0143	0.9367	662	-0.0271	0.0081	91	0.0013	0.9597	96	-0.0240	0.4871
PPOX	ILMN_1673798	0.0143	0.9367	718	-0.0533	0.0082	152	-0.0702	0.1563	96	0.0420	0.3653
ICAM2	ILMN_1786823	0.0144	0.9367	718	-0.0286	0.2016	152	-0.1489	0.0304	96	-0.0904	0.0596
LOC645515	ILMN_3285959	0.0146	0.9367	716	0.0656	0.1676	152	0.1429	0.0826	96	0.1075	0.0333
ALPK1	ILMN_2078697	0.0146	0.9367	717	0.0779	0.0238	152	0.1136	0.2203	96	0.0248	0.7143
NAAA	ILMN_2391512	0.0147	0.9367	717	-0.1142	0.0012	150	0.1861	0.0377	96	-0.1228	0.0739
	ILMN_1865764	0.0147	0.9367	718	0.0588	0.0068	151	-0.0264	0.7064	96	0.0475	0.2536

YWHAH	ILMN_1728512	0.0148	0.9367	718	-0.0695	0.0036	152	-0.0001	0.9989	96	-0.0059	0.9190
RNF149	ILMN_2112524	0.0148	0.9367	718	0.0686	0.0429	152	0.1403	0.0849	96	0.0203	0.7143
SFRS1	ILMN_1795341	0.0148	0.9367	718	0.0430	0.0205	152	0.1154	0.0893	96	-0.0154	0.6862
SLC3A2	ILMN_1726456	0.0153	0.9367	717	-0.0385	0.0876	142	-0.0909	0.1025	96	-0.0562	0.1845
RHOT1	ILMN_2338480	0.0154	0.9367	712	0.0556	0.0713	131	0.1239	0.0109	96	0.0014	0.9778
LOC646562	ILMN_1665970	0.0154	0.9367	666	-0.1528	0.0239	88	-0.0815	0.3900	96	-0.0479	0.4504
XRN2	ILMN_1727617	0.0154	0.9367	712	0.0583	0.0226	148	0.0873	0.1474	96	-0.0012	0.9780
MKLN1	ILMN_1742578	0.0155	0.9367	718	0.0594	0.0243	152	0.1223	0.2282	96	0.0179	0.7345
SUSD1	ILMN_1709750	0.0157	0.9367	718	-0.0581	0.0107	150	0.0253	0.6136	96	-0.0756	0.1007
DEDD	ILMN_2313074	0.0158	0.9367	718	-0.0336	0.0494	152	-0.0770	0.1068	96	-0.0283	0.5556
PPWD1	ILMN_2223380	0.0158	0.9367	709	0.0708	0.0357	125	0.1172	0.0725	96	0.0038	0.9301
ACSL5	ILMN_2370882	0.0158	0.9367	687	-0.0424	0.0563	89	-0.0109	0.7448	96	-0.0877	0.0297
RPL14	ILMN_1726460	0.0159	0.9367	718	0.0777	0.0748	152	0.0918	0.3846	96	0.1565	0.0431
SH3GLB1	ILMN_1766045	0.0159	0.9367	718	0.0447	0.0947	152	0.1478	0.0229	96	0.0282	0.5810
FAM165B	ILMN_3238845	0.0162	0.9367	707	-0.0195	0.0571	137	-0.0339	0.2419	96	-0.0552	0.1972
DDX59	ILMN_1748077	0.0162	0.9367	717	0.0826	0.0056	147	0.0016	0.9809	96	0.0166	0.7320
C21orf70	ILMN_2104924	0.0164	0.9367	699	-0.0214	0.0362	129	-0.0573	0.0173	96	0.0217	0.5237
SASH3	ILMN_1697554	0.0164	0.9367	718	-0.0455	0.0703	152	-0.0986	0.0887	96	-0.0451	0.3925
C2orf34	ILMN_1738099	0.0165	0.9367	713	-0.0386	0.0431	83	-0.0529	0.1059	96	-0.0277	0.5400
	ILMN_1856861	0.0166	0.9367	690	0.0616	0.0051	136	0.0232	0.5455	96	-0.0192	0.6301
PRPF19	ILMN_1769545	0.0167	0.9367	710	-0.0525	0.0401	144	-0.0725	0.1643	96	-0.0319	0.5681
	ILMN_1904135	0.0168	0.9367	695	0.0595	0.0100	110	0.0414	0.3087	96	-0.0140	0.7104
GIPC1	ILMN_1796177	0.0169	0.9367	716	-0.0555	0.0290	144	0.0120	0.7977	96	-0.1129	0.0268
TMEM185A	ILMN_2140389	0.0171	0.9367	717	-0.0276	0.1158	146	-0.0824	0.0504	96	-0.0526	0.2489
C3orf37	ILMN_1815682	0.0172	0.9367	716	-0.0429	0.0585	152	-0.1085	0.0209	96	0.0099	0.8487
DUSP14	ILMN_1666546	0.0172	0.9367	696	-0.0212	0.0942	90	-0.0551	0.0128	96	-0.0259	0.5373
TMX3	ILMN_2047206	0.0173	0.9367	713	0.1052	0.0021	135	0.0471	0.5173	96	-0.0590	0.1742
OGDH	ILMN_1733869	0.0175	0.9367	715	-0.0467	0.0913	150	-0.1061	0.0865	96	-0.0641	0.2736
KIAA1468	ILMN_1798346	0.0176	0.9367	712	0.1132	0.0020	100	0.0001	0.9986	96	-0.0370	0.4360
C12orf41	ILMN_1713189	0.0176	0.9367	718	0.0496	0.0094	150	0.0462	0.5174	96	-0.0034	0.9524
TFIP11	ILMN_1695000	0.0177	0.9367	718	0.0567	0.0499	148	0.0370	0.4685	96	0.0868	0.1143
TTC38	ILMN_2059886	0.0179	0.9367	718	-0.0644	0.0935	152	-0.1774	0.0285	96	-0.0332	0.6206
CAPRIN2	ILMN_1681118	0.0179	0.9367	714	0.0632	0.0120	136	0.0286	0.4871	96	0.0035	0.9333
LCMT1	ILMN_1688452	0.0179	0.9367	698	-0.0219	0.0363	114	-0.0120	0.6687	96	-0.0695	0.1373
MAF	ILMN_1719543	0.0180	0.9367	712	-0.0509	0.0049	100	0.0123	0.6736	96	-0.0180	0.7133
BCL7B	ILMN_2367275	0.0181	0.9367	715	-0.0467	0.0111	107	-0.0136	0.6409	96	-0.0086	0.8399
CYB561	ILMN_2378376	0.0181	0.9367	715	-0.0633	0.0020	137	-0.0044	0.8974	96	0.0430	0.3935
RNF13	ILMN_2339748	0.0181	0.9367	718	0.0829	0.0109	151	0.1261	0.2619	96	-0.0309	0.5811
HNRPDL	ILMN_1653432	0.0183	0.9367	718	0.0748	0.0210	141	0.1070	0.1396	96	-0.0165	0.7410
CCNL1	ILMN_2094776	0.0186	0.9367	717	0.0725	0.0413	142	0.1651	0.0545	96	-0.0109	0.8684
PCGF1	ILMN_1757956	0.0186	0.9367	616	-0.0190	0.0652	115	-0.0213	0.5147	96	-0.0816	0.0682
PAFAH2	ILMN_1682919	0.0189	0.9367	685	-0.0240	0.1246	146	-0.1142	0.0282	96	-0.0410	0.4194
OGFOD1	ILMN_1677953	0.0191	0.9367	711	-0.0280	0.1038	150	-0.0772	0.0375	96	-0.0274	0.4865

UBXN4	ILMN_1781097	0.0193	0.9367	718	0.0848	0.0063	152	0.0627	0.5344	96	-0.0282	0.6153
AP3D1	ILMN_1764945	0.0194	0.9367	708	-0.0462	0.0156	128	-0.0088	0.7913	96	-0.0362	0.4640
HIC2	ILMN_1652762	0.0195	0.9367	716	-0.0270	0.0674	146	-0.0331	0.1844	96	-0.0403	0.2831
GTSCR1	ILMN_1708004	0.0195	0.9367	542	-0.0307	0.0852	79	-0.0730	0.0364	96	-0.0336	0.4996
RNF220	ILMN_1694504	0.0195	0.9367	718	-0.0319	0.0600	151	-0.0459	0.2954	96	-0.0519	0.2034
TRIM22	ILMN_1779252	0.0196	0.9367	717	0.1103	0.0183	145	0.1479	0.2568	96	-0.0124	0.8872
DICER1	ILMN_1772692	0.0196	0.9367	717	0.0810	0.0133	145	0.0669	0.4814	96	0.0048	0.9470
RBMS1	ILMN_1666444	0.0196	0.9367	718	0.0468	0.0333	152	0.0814	0.1822	96	0.0108	0.8147
LOC653778	ILMN_1707434	0.0196	0.9367	718	0.0369	0.2919	152	0.1948	0.0470	96	0.2200	0.0188
ZDHHC6	ILMN_1739659	0.0196	0.9367	718	0.0669	0.0027	151	-0.0205	0.6942	96	0.0009	0.9844
ZFP36L1	ILMN_1675448	0.0196	0.9367	718	0.0442	0.1297	152	0.2136	0.0541	96	0.0804	0.2456
WDR75	ILMN_1801869	0.0197	0.9367	718	0.0852	0.0036	151	0.0727	0.3683	96	-0.0607	0.1742
PLXNB2	ILMN_1763447	0.0198	0.9367	717	-0.0522	0.1716	149	-0.1863	0.0182	96	-0.0657	0.3070
DNAJC5	ILMN_1789642	0.0198	0.9367	711	-0.0380	0.0341	152	-0.0581	0.2612	96	-0.0205	0.6145
CDKN1B	ILMN_1722811	0.0200	0.9367	718	0.0489	0.0722	152	0.1342	0.1419	96	0.0447	0.3477
ACSL1	ILMN_1684585	0.0201	0.9367	718	0.0645	0.1027	152	0.2237	0.0591	96	0.0706	0.3896
SHOC2	ILMN_2158242	0.0201	0.9367	718	0.0529	0.0820	152	0.1456	0.1045	96	0.0475	0.3698
CRIP1	ILMN_1656920	0.0202	0.9367	718	-0.0447	0.0880	152	-0.0647	0.3583	96	-0.1635	0.0613
GUCA2A	ILMN_1763749	0.0202	0.9367	630	-0.0428	0.3597	94	-0.0312	0.5231	96	-0.2572	4.06E-05
P4HB	ILMN_1719303	0.0202	0.9367	718	-0.0307	0.1258	152	-0.0466	0.4148	96	-0.1301	0.0134
LOC730235	ILMN_3231621	0.0203	0.9367	718	0.0548	0.1732	152	0.1173	0.3375	96	0.2207	0.0058
	ILMN_1913678	0.0203	0.9367	718	0.0756	0.0280	151	0.0494	0.6398	96	0.0683	0.2787
IL4R	ILMN_1652185	0.0205	0.9367	718	0.0986	0.0053	152	0.0192	0.8329	96	-0.0152	0.8229
STX3	ILMN_1659544	0.0206	0.9367	718	0.0712	0.0193	151	0.0986	0.2609	96	-0.0085	0.8850
HLA-DPB1	ILMN_1749070	0.0206	0.9367	716	0.1168	0.0050	130	0.0385	0.6243	96	-0.0479	0.5083
ZNF689	ILMN_1759008	0.0206	0.9367	718	-0.0566	0.0161	152	-0.0559	0.3522	96	0.0039	0.9301
ACSL3	ILMN_2360705	0.0209	0.9367	716	0.0579	0.0391	123	0.0729	0.1146	96	0.0054	0.9156
FAM126B	ILMN_1779486	0.0211	0.9367	718	0.0762	0.0721	152	0.1471	0.1764	96	0.0688	0.3083
HBB	ILMN_2100437	0.0212	0.9367	718	0.0259	0.0365	152	0.0278	0.5021	96	0.0402	0.2851
	ILMN_1864166	0.0212	0.9367	708	-0.0324	0.0887	140	-0.0554	0.1179	96	-0.0530	0.3024
TEX261	ILMN_1692272	0.0212	0.9367	704	-0.0290	0.0145	101	-0.0097	0.6867	96	-0.0138	0.7391
LOC100131387	ILMN_3293676	0.0213	0.9367	718	0.0493	0.0534	152	0.1212	0.2443	96	0.0727	0.3810
AASDH	ILMN_1784269	0.0216	0.9367	711	0.0644	0.0030	103	0.0047	0.9004	96	-0.0283	0.4124
INPP1	ILMN_1667239	0.0217	0.9367	711	-0.0374	0.0368	135	-0.0294	0.4010	96	-0.0351	0.4081
DDX60L	ILMN_3243928	0.0217	0.9367	712	0.0792	0.0438	144	0.1416	0.1215	96	0.0103	0.8720
RPL18	ILMN_2230624	0.0219	0.9367	718	-0.0208	0.0699	152	-0.0459	0.1933	96	-0.0460	0.3187
CD320	ILMN_2115633	0.0219	0.9367	632	-0.0312	0.1272	107	-0.0521	0.2254	96	-0.0982	0.0627
LOC647000	ILMN_1703692	0.0219	0.9367	718	-0.0433	0.1121	152	-0.1120	0.1028	96	-0.0523	0.2342
LOC100128771	ILMN_3263694	0.0219	0.9367	657	0.1118	0.0092	135	0.0152	0.7615	96	0.0035	0.9200
CDKN1C	ILMN_1718565	0.0220	0.9367	663	-0.0700	0.0309	84	-0.0617	0.2050	96	-0.0176	0.8209
SMARCD1	ILMN_1728845	0.0220	0.9367	718	-0.0505	0.0441	152	-0.0233	0.7588	96	-0.0619	0.0912
CMTM8	ILMN_1710124	0.0220	0.9367	713	0.0766	0.0267	143	0.1469	0.0559	96	-0.0500	0.3995

CROP	ILMN_2299072	0.0221	0.9367	718	0.0786	0.0336	149	0.1138	0.4086	96	0.0375	0.4662
P2RX1	ILMN_1758529	0.0225	0.9367	714	-0.0501	0.0765	148	-0.0793	0.3056	96	-0.0903	0.1550
ALDH3B1	ILMN_1728662	0.0226	0.9367	716	-0.0641	0.0297	148	-0.0411	0.5711	96	-0.0447	0.3746
NAG18	ILMN_2090949	0.0227	0.9367	718	0.0301	0.0322	152	0.0083	0.8434	96	0.0551	0.1488
BCCIP	ILMN_1771966	0.0227	0.9367	705	0.0558	0.0147	144	0.0187	0.7069	96	0.0201	0.6938
APEH	ILMN_1718023	0.0228	0.9367	718	-0.0243	0.1677	152	-0.0934	0.0812	96	-0.0735	0.1157
HES4	ILMN_1653466	0.0228	0.9367	717	-0.1003	0.0689	151	-0.2540	0.0498	96	-0.0075	0.9221
DHRS4	ILMN_1762666	0.0228	0.9367	686	-0.0252	0.0569	123	-0.0528	0.1053	96	-0.0226	0.7108
DCTD	ILMN_1802456	0.0230	0.9367	718	-0.0273	0.1090	151	-0.0136	0.7523	96	-0.1348	0.0060
H3F3B	ILMN_1695706	0.0231	0.9367	718	0.0453	0.0177	152	0.0795	0.3028	96	-0.0132	0.7979
CYC1	ILMN_1815115	0.0231	0.9367	718	-0.0302	0.1521	152	-0.0708	0.2217	96	-0.1032	0.0386
PIP5K1C	ILMN_1668514	0.0234	0.9367	717	-0.0622	0.0555	112	-0.0672	0.2293	96	-0.0429	0.4173
SNORD12C	ILMN_3249286	0.0236	0.9367	597	0.0343	0.0086	93	0.0021	0.9426	96	0.0064	0.8867
TIPRL	ILMN_2249288	0.0237	0.9367	667	0.0633	0.0126	90	-0.0065	0.8441	96	0.0261	0.5123
CCNT2	ILMN_1722522	0.0237	0.9367	705	0.0762	0.0168	113	0.0375	0.5865	96	0.0100	0.8269
SKAP2	ILMN_1657129	0.0237	0.9367	718	0.0697	0.0245	152	0.1017	0.2745	96	-0.0023	0.9716
TGFBR2	ILMN_1726245	0.0240	0.9367	718	0.0433	0.0393	152	0.1203	0.0831	96	-0.0179	0.7324
C14orf135	ILMN_1680781	0.0240	0.9367	714	0.0719	0.0196	135	0.0495	0.3883	96	0.0002	0.9966
ABHD12	ILMN_2245305	0.0242	0.9367	702	-0.0245	0.1167	126	-0.0405	0.0875	96	-0.0379	0.2716
GNA15	ILMN_1773963	0.0247	0.9367	717	-0.0350	0.1231	149	-0.1327	0.0267	96	-0.0204	0.6541
SSR4	ILMN_1680403	0.0247	0.9367	718	-0.0188	0.3726	152	-0.1409	0.0114	96	-0.0944	0.0696
PTPRA	ILMN_2411794	0.0248	0.9367	718	-0.0237	0.2278	152	-0.1046	0.0490	96	-0.0714	0.0979
ALDH2	ILMN_1793859	0.0249	0.9367	718	-0.0670	0.0347	147	-0.0187	0.7878	96	-0.0860	0.1943
BRI3BP	ILMN_1693410	0.0250	0.9367	718	-0.0550	0.0323	152	-0.0976	0.2556	96	-0.0073	0.8902
RNF125	ILMN_1747192	0.0253	0.9367	645	-0.0331	0.0317	93	-0.0216	0.3697	96	-0.0140	0.6675
RANBP1	ILMN_1721457	0.0254	0.9367	709	-0.0416	0.0497	128	-0.0079	0.8362	96	-0.0917	0.0885
LOC727865	ILMN_3276209	0.0254	0.9367	718	0.1299	0.0118	152	0.1034	0.3758	96	-0.0728	0.5481
GSTP1	ILMN_1679809	0.0255	0.9367	718	-0.0372	0.0580	152	-0.0989	0.1270	96	-0.0152	0.7687
KLHL26	ILMN_1805330	0.0257	0.9367	702	-0.0311	0.0875	75	-0.0435	0.2601	96	-0.0608	0.1597
WDR19	ILMN_1655117	0.0257	0.9367	713	0.0413	0.0690	117	0.0516	0.0740	96	0.0105	0.7590
ZNF137	ILMN_1723542	0.0258	0.9367	718	0.0484	0.0725	118	0.0377	0.0847	96	0.0156	0.6653
PGS1	ILMN_2075051	0.0259	0.9367	718	0.0443	0.0718	152	0.1743	0.0249	96	-0.0220	0.7128
MRPS2	ILMN_1815043	0.0259	0.9367	715	-0.0382	0.0365	132	-0.0383	0.3074	96	-0.0171	0.7042
PKP2	ILMN_2386973	0.0260	0.9367	657	-0.1961	0.0114	80	0.0970	0.6096	96	-0.0357	0.4911
BEST1	ILMN_1718982	0.0260	0.9367	717	0.0701	0.0221	150	0.0472	0.4301	96	0.0062	0.9021
PPP6C	ILMN_1664921	0.0262	0.9367	718	-0.0236	0.1771	152	-0.0915	0.0199	96	-0.0250	0.4579
TMEM188	ILMN_1653134	0.0262	0.9367	716	0.0565	0.0299	146	0.1338	0.1287	96	-0.0240	0.6391
METT11D1	ILMN_2276811	0.0263	0.9367	707	0.0542	0.0081	126	0.0131	0.6164	96	-0.0216	0.5866
MYO1G	ILMN_1692295	0.0267	0.9367	718	-0.0473	0.1525	152	-0.1216	0.1501	96	-0.0963	0.1066
	ILMN_1889215	0.0267	0.9367	476	-0.0313	0.0454	118	-0.0242	0.4322	96	-0.0391	0.3834
TAF7	ILMN_1759460	0.0268	0.9367	718	0.0767	0.0456	149	0.2286	0.0217	96	-0.0581	0.3159
PAN3	ILMN_1681304	0.0269	0.9367	718	0.0541	0.0389	152	0.1339	0.1557	96	-0.0053	0.9172
RTN1	ILMN_1756928	0.0271	0.9367	712	-0.1004	0.0144	129	0.0125	0.8185	96	-0.0522	0.4191

	ILMN_1836218	0.0271	0.9367	699	0.0366	0.0088	88	0.0020	0.9500	96	-0.0050	0.8956
METTL14	ILMN_2124523	0.0271	0.9367	602	0.0216	0.0139	104	0.0109	0.6410	96	0.0009	0.9792
TIGD5	ILMN_1705774	0.0274	0.9367	712	-0.0406	0.0111	139	-0.0138	0.6987	96	0.0071	0.8815
PHPT1	ILMN_1676611	0.0274	0.9367	716	-0.0349	0.1076	152	-0.0948	0.0740	96	-0.0338	0.5135
	ILMN_1893511	0.0275	0.9367	714	0.0514	0.0340	145	0.1005	0.1484	96	-0.0132	0.7658
B2M	ILMN_2148459	0.0275	0.9367	718	0.0251	0.1739	152	0.1332	0.0700	96	0.0697	0.1927
CST3	ILMN_1800354	0.0275	0.9367	718	-0.0525	0.0878	152	-0.0484	0.5120	96	-0.0973	0.0715
FCGR1A	ILMN_2176063	0.0276	0.9367	714	0.1258	0.0182	147	0.0628	0.5834	96	0.0137	0.8839
IDI1	ILMN_1755075	0.0276	0.9367	717	0.0624	0.0528	139	0.1073	0.1109	96	0.0017	0.9758
BAZ2B	ILMN_1720850	0.0277	0.9367	718	0.0964	0.0217	152	0.0742	0.5102	96	0.0109	0.8525
LOC388588	ILMN_1749834	0.0277	0.9367	718	0.0277	0.7841	152	0.8266	0.0016	96	0.5815	0.0099
ATP6V1C1	ILMN_1659801	0.0278	0.9367	709	0.0522	0.0065	145	0.0415	0.4551	96	-0.0497	0.2761
TIMP1	ILMN_1711566	0.0278	0.9367	718	-0.0588	0.0472	152	-0.1065	0.2179	96	-0.0246	0.7589
CCL5	ILMN_2098126	0.0279	0.9367	718	-0.0779	0.0039	152	-0.0349	0.6710	96	0.0878	0.2485
RAB33B	ILMN_1727738	0.0280	0.9367	714	0.0855	0.0325	143	-0.0476	0.6266	96	0.1117	0.0463
EHD4	ILMN_1720083	0.0281	0.9367	712	-0.0340	0.0907	146	-0.0201	0.6633	96	-0.1070	0.0374
CIRH1A	ILMN_1796235	0.0282	0.9367	718	-0.0152	0.4339	151	-0.1114	0.0141	96	-0.0858	0.0413
RAP2C	ILMN_1773561	0.0283	0.9367	718	0.0424	0.1174	152	0.1130	0.1446	96	0.0539	0.2512
CSK	ILMN_1754121	0.0283	0.9367	718	-0.0585	0.0118	152	-0.0394	0.5789	96	0.0151	0.7455
USP8	ILMN_1715188	0.0284	0.9367	710	0.0417	0.0680	147	0.0497	0.3894	96	0.0495	0.2375
FUCA2	ILMN_1744914	0.0285	0.9367	712	-0.0270	0.0565	141	-0.0425	0.2055	96	-0.0230	0.6436
UNC84B	ILMN_2099301	0.0286	0.9367	718	-0.0403	0.0677	152	-0.0757	0.2118	96	-0.0356	0.4958
RCSD1	ILMN_1749006	0.0286	0.9367	718	-0.0261	0.1745	152	-0.1135	0.0419	96	-0.0415	0.3310
ZNF451	ILMN_1706734	0.0287	0.9367	713	0.0676	0.0103	146	0.0149	0.8376	96	-0.0021	0.9658
TMBIM6	ILMN_1693311	0.0287	0.9367	718	-0.0369	0.0785	152	-0.0526	0.4675	96	-0.0806	0.1294
ZNF550	ILMN_1760102	0.0288	0.9367	712	0.0578	0.0061	94	0.0002	0.9930	96	-0.0188	0.6319
BIRC2	ILMN_1768194	0.0291	0.9367	694	0.0560	0.0142	151	0.0598	0.4033	96	-0.0223	0.6094
GORASP2	ILMN_1748018	0.0292	0.9367	713	-0.0354	0.1942	144	-0.0829	0.0616	96	-0.0757	0.1789
NKIRAS2	ILMN_1653404	0.0292	0.9367	716	-0.0358	0.0541	149	-0.0551	0.2816	96	-0.0253	0.5478
LOC644590	ILMN_1664233	0.0295	0.9367	713	-0.0523	0.0603	133	-0.0405	0.3957	96	-0.0547	0.3181
ZSWIM1	ILMN_1812856	0.0297	0.9367	718	-0.0376	0.1251	152	-0.1830	0.0082	96	0.0147	0.7460
OMA1	ILMN_1670079	0.0298	0.9367	716	0.0616	0.0237	150	0.0513	0.3973	96	-0.0029	0.9560
UHMK1	ILMN_2096012	0.0299	0.9367	711	-0.0517	0.1493	152	-0.1656	0.0638	96	-0.0363	0.3649
LRRC33	ILMN_1746148	0.0301	0.9367	712	-0.0679	0.0317	146	-0.0315	0.6247	96	-0.0362	0.4929
DHPS	ILMN_2326997	0.0301	0.9367	718	-0.0356	0.0549	152	-0.0320	0.5748	96	-0.0527	0.2211
PPA1	ILMN_1805827	0.0302	0.9367	718	0.0743	0.0394	152	0.2385	0.0181	96	-0.0973	0.1534
STARD5	ILMN_1784364	0.0302	0.9367	700	-0.0253	0.0607	78	-0.0309	0.3364	96	-0.0267	0.3705
CFI	ILMN_1727815	0.0302	0.9367	690	-0.0215	0.0717	107	-0.0541	0.0765	96	-0.0087	0.8400
C5orf15	ILMN_1695917	0.0302	0.9367	718	0.0636	0.0048	152	-0.0188	0.8346	96	-0.0108	0.7931
LIAS	ILMN_2393341	0.0303	0.9367	557	-0.0223	0.1403	100	-0.0467	0.0291	96	-0.0192	0.5803
CCNF	ILMN_1773119	0.0304	0.9367	669	-0.0147	0.2754	101	-0.0773	0.0044	96	-0.0350	0.3239
FCGR2A	ILMN_1666932	0.0305	0.9367	718	0.0511	0.0536	152	0.0578	0.3989	96	0.0485	0.4078

TCEB2	ILMN_2377185	0.0306	0.9367	691	-0.0314	0.0167	79	-0.0228	0.3583	96	0.0162	0.6347
TTYH3	ILMN_1692731	0.0307	0.9367	718	-0.0555	0.0356	146	-0.0144	0.7277	96	-0.0473	0.3403
ARHGAP15	ILMN_2208413	0.0308	0.9367	718	0.0353	0.0692	152	0.1282	0.0753	96	-0.0029	0.9581
IL1RAP	ILMN_1686884	0.0308	0.9367	718	0.0770	0.0765	147	0.0673	0.4176	96	0.0774	0.1981
LAT	ILMN_2404625	0.0309	0.9367	718	-0.0438	0.0425	152	-0.0770	0.2395	96	-0.0048	0.9074
EXOSC6	ILMN_1670218	0.0311	0.9367	717	-0.0241	0.2820	144	-0.0948	0.0354	96	-0.0765	0.1120
C14orf138	ILMN_1781102	0.0311	0.9367	706	0.0688	0.0440	78	0.0691	0.1424	96	0.0014	0.9749
LOC646044	ILMN_3209973	0.0311	0.9367	717	-0.0359	0.1467	151	-0.0831	0.1107	96	-0.0578	0.2450
MEMO1	ILMN_1741599	0.0312	0.9367	717	0.0367	0.0138	151	-0.0146	0.7400	96	0.0342	0.4139
EXOC3	ILMN_1789419	0.0312	0.9367	715	-0.0388	0.1012	145	-0.0720	0.1389	96	-0.0416	0.4213
SCRN1	ILMN_1756439	0.0313	0.9367	717	-0.0329	0.1899	134	-0.1114	0.0025	96	0.0053	0.9204
CMTM6	ILMN_1696494	0.0314	0.9367	718	0.0476	0.0836	152	0.2314	0.0351	96	-0.0162	0.7993
LOC729439	ILMN_3244192	0.0317	0.9367	718	0.0392	0.0598	152	-0.0058	0.8932	96	0.1293	0.0326
LASP1	ILMN_1665909	0.0317	0.9367	718	-0.0528	0.0389	152	-0.0576	0.2991	96	-0.0087	0.8763
CHMP4B	ILMN_1771233	0.0317	0.9367	718	-0.0642	0.0038	152	0.0197	0.6608	96	0.0120	0.8000
LOC285741	ILMN_3216336	0.0318	0.9367	718	0.0612	0.1229	152	0.2738	0.0582	96	0.0416	0.6096
CBWD5	ILMN_2150352	0.0318	0.9367	704	0.0416	0.0705	99	0.0508	0.1599	96	0.0239	0.5934
SHC1	ILMN_1721022	0.0321	0.9367	718	-0.0078	0.7121	152	-0.1844	0.0063	96	-0.0843	0.0080
VDAC1	ILMN_2175601	0.0323	0.9367	718	-0.0357	0.0279	152	-0.0099	0.8256	96	-0.0341	0.4258
RPL9	ILMN_2408415	0.0324	0.9367	718	0.0764	0.0312	152	0.1556	0.3077	96	-0.0109	0.9299
MGC3196	ILMN_2263144	0.0326	0.9367	702	-0.0144	0.3210	133	-0.0698	0.0893	96	-0.1084	0.0236
LOC644132	ILMN_3237368	0.0326	0.9367	715	-0.0255	0.1337	139	-0.0379	0.1828	96	-0.0561	0.1864
RGL4	ILMN_1663422	0.0326	0.9367	718	0.0549	0.1041	152	0.0806	0.2898	96	0.0901	0.1930
HELQ	ILMN_1720440	0.0327	0.9367	701	0.0352	0.0499	95	0.0484	0.1266	96	-0.0006	0.9849
TPM4	ILMN_1653180	0.0328	0.9367	712	-0.0354	0.1808	140	-0.1189	0.0569	96	-0.0541	0.2901
ISG20L2	ILMN_2090397	0.0329	0.9367	718	-0.0460	0.0845	152	-0.0630	0.4074	96	-0.0715	0.1926
ARPC2	ILMN_1810200	0.0330	0.9367	702	-0.1206	0.0033	117	0.0001	0.9991	96	0.0504	0.2806
TAZ	ILMN_1776679	0.0332	0.9367	708	-0.0716	0.0162	91	0.0109	0.8026	96	-0.0193	0.6138
	ILMN_1867588	0.0333	0.9367	603	0.0280	0.0843	100	0.0802	0.0400	96	-0.0006	0.9899
CD5	ILMN_1753112	0.0334	0.9367	717	-0.0467	0.1058	145	-0.0050	0.9362	96	-0.1423	0.0126
JMJD1C	ILMN_2410742	0.0336	0.9367	717	0.0702	0.0876	138	0.2169	0.0253	96	-0.0260	0.7140
LRRFIP2	ILMN_1707156	0.0336	0.9367	718	0.0323	0.1377	149	0.0667	0.1026	96	0.0372	0.3508
SLAMF6	ILMN_2196078	0.0336	0.9367	718	-0.0381	0.0535	152	-0.0825	0.2000	96	-0.0067	0.8878
KCTD13	ILMN_1786843	0.0336	0.9367	718	-0.1559	0.0085	149	0.0598	0.4220	96	-0.0365	0.4094
PLA2G15	ILMN_1756910	0.0337	0.9367	705	-0.0237	0.1249	93	-0.0406	0.1505	96	-0.0398	0.2332
COPA	ILMN_1811615	0.0338	0.9367	718	-0.0295	0.1626	152	-0.0865	0.1274	96	-0.0550	0.1967
SCAND1	ILMN_1794230	0.0339	0.9367	715	-0.0427	0.0429	152	-0.0605	0.3499	96	-0.0148	0.7579
FYCO1	ILMN_1709032	0.0339	0.9367	705	-0.0311	0.0685	151	-0.0629	0.1650	96	-0.0129	0.7706
CDC123	ILMN_1678605	0.0340	0.9367	718	0.0584	0.0382	151	0.1096	0.0840	96	-0.0374	0.4149
SNHG11	ILMN_1791884	0.0340	0.9367	718	-0.0426	0.0726	148	-0.0247	0.5222	96	-0.0492	0.1933
IDH3B	ILMN_2373632	0.0341	0.9367	718	-0.0263	0.1725	148	-0.0769	0.1597	96	-0.0649	0.1273
MUL1	ILMN_3235168	0.0342	0.9367	718	-0.0292	0.1060	152	-0.0800	0.1086	96	-0.0256	0.5676
LINS1	ILMN_2338197	0.0344	0.9367	718	0.0451	0.0660	147	0.0610	0.1347	96	0.0051	0.9115

RGS12	ILMN_1722834	0.0345	0.9367	701	-0.0322	0.1326	98	-0.0564	0.1064	96	-0.0411	0.2980
PPP2R2D	ILMN_1778587	0.0347	0.9367	718	-0.0290	0.0415	150	-0.0155	0.6382	96	-0.0327	0.4112
SLPI	ILMN_2114720	0.0347	0.9367	661	0.0936	0.0609	126	0.0808	0.3537	96	0.0490	0.4788
RALA	ILMN_1755364	0.0347	0.9367	718	0.0402	0.0060	152	0.0114	0.8351	96	-0.0311	0.4319
CHST13	ILMN_1734707	0.0348	0.9367	717	-0.1178	0.0207	148	-0.1192	0.2699	96	0.0714	0.4700
CENPB	ILMN_1664028	0.0349	0.9367	718	-0.0446	0.0693	152	-0.1044	0.2145	96	-0.0235	0.6482
DCK	ILMN_1651433	0.0349	0.9367	713	0.0709	0.0677	127	0.1391	0.1001	96	0.0005	0.9937
MTA2	ILMN_1773763	0.0349	0.9367	713	-0.0511	0.0242	148	-0.0631	0.3127	96	0.0221	0.6559
FAM48A	ILMN_1669555	0.0349	0.9367	717	0.0713	0.0182	147	0.0019	0.9658	96	0.0202	0.6477
RNF146	ILMN_1685679	0.0350	0.9367	709	0.0499	0.0711	107	0.1101	0.0395	96	-0.0122	0.7687
PHF20L1	ILMN_1732985	0.0350	0.9367	717	0.0823	0.0355	147	0.1076	0.2912	96	-0.0055	0.9266
MYO9A	ILMN_2173919	0.0351	0.9367	675	-0.0209	0.1330	122	-0.0963	0.0170	96	-0.0016	0.9731
MAZ	ILMN_1677997	0.0351	0.9367	716	0.0788	0.0236	150	0.0019	0.9769	96	0.0333	0.4553
SELL	ILMN_1724422	0.0351	0.9367	718	0.0443	0.1233	152	0.1764	0.0384	96	0.0114	0.8741
C1orf166	ILMN_1675055	0.0352	0.9367	717	-0.0414	0.0194	139	-0.0046	0.8994	96	-0.0160	0.7043
AKAP11	ILMN_1693220	0.0353	0.9367	717	0.0522	0.0644	142	0.0857	0.1587	96	0.0074	0.8683
CXorf48	ILMN_1808984	0.0353	0.9367	687	-0.1041	0.0241	136	-0.0640	0.3284	96	0.0209	0.6898
NUP50	ILMN_1725612	0.0354	0.9367	714	0.0412	0.0445	136	0.0442	0.2369	96	0.0004	0.9935
TMEM71	ILMN_1674402	0.0355	0.9367	718	0.0470	0.1244	152	0.1268	0.1961	96	0.0683	0.2553
SMAD7	ILMN_2203891	0.0356	0.9367	697	-0.0299	0.0258	97	-0.0043	0.8870	96	-0.0189	0.5977
COMMD5	ILMN_2300396	0.0356	0.9367	715	-0.0404	0.0361	149	-0.0338	0.4693	96	-0.0156	0.7536
IVNS1ABP	ILMN_1717877	0.0357	0.9367	718	0.0433	0.0667	152	0.1064	0.1971	96	0.0183	0.7502
STK25	ILMN_1668090	0.0360	0.9367	718	-0.0250	0.1388	151	-0.0565	0.1758	96	-0.0512	0.2323
VCPIP1	ILMN_1682180	0.0361	0.9367	718	0.0763	0.0189	150	0.0759	0.4198	96	-0.0279	0.6207
C11orf17	ILMN_1752988	0.0361	0.9367	717	-0.0667	0.0044	139	-0.0099	0.7852	96	0.0578	0.2234
TRIM33	ILMN_1682316	0.0362	0.9367	718	0.0690	0.0199	151	0.0886	0.4076	96	-0.0215	0.6385
UNC45A	ILMN_1726434	0.0364	0.9367	718	-0.0315	0.2448	152	-0.1581	0.0258	96	-0.0430	0.3468
LOC653773	ILMN_1659405	0.0367	0.9367	718	0.0689	0.1269	152	0.1364	0.3796	96	0.1652	0.1008
FAM60A	ILMN_3272603	0.0367	0.9367	718	0.0546	0.0433	149	0.1092	0.1782	96	-0.0128	0.7676
TRIM25	ILMN_1813625	0.0369	0.9367	718	0.1084	0.0065	152	0.0138	0.8469	96	-0.0447	0.4374
KLHL6	ILMN_1768814	0.0371	0.9367	707	0.0476	0.0288	125	0.0278	0.4476	96	-0.0016	0.9752
TRPV2	ILMN_2049536	0.0371	0.9367	717	-0.0401	0.1285	150	-0.1080	0.0666	96	-0.0203	0.6572
N4BP2L2	ILMN_3234089	0.0374	0.9367	718	0.0814	0.0220	150	0.0278	0.7656	96	0.0115	0.8038
FAM190B	ILMN_2129388	0.0375	0.9367	706	0.0576	0.0091	140	0.0595	0.3412	96	-0.0621	0.1558
SDHA	ILMN_2051232	0.0375	0.9367	717	-0.0352	0.1548	140	-0.0780	0.1202	96	-0.0409	0.2828
TESK2	ILMN_1654370	0.0376	0.9367	712	0.0351	0.0241	122	0.0311	0.1752	96	-0.0368	0.3618
DHX37	ILMN_2192683	0.0377	0.9367	718	-0.0224	0.3644	149	-0.1301	0.0099	96	-0.0519	0.2405
LOC730534	ILMN_1689327	0.0381	0.9367	718	-0.0404	0.0205	152	0.0539	0.3497	96	-0.0921	0.0877
GPI	ILMN_2173451	0.0381	0.9367	718	-0.0317	0.2749	151	-0.1310	0.0571	96	-0.0638	0.1360
EWSR1	ILMN_1697735	0.0381	0.9367	716	0.0848	0.0154	152	0.0441	0.5550	96	-0.0206	0.6160
NINJ1	ILMN_1815086	0.0382	0.9367	718	-0.0601	0.0315	152	-0.0393	0.6176	96	-0.0257	0.7241
PDE7A	ILMN_2278819	0.0384	0.9367	691	0.0540	0.0210	88	-0.0069	0.8175	96	0.0214	0.5780

TMEM194A	ILMN_3306440	0.0385	0.9367	690	0.0432	0.0156	105	0.0255	0.3981	96	-0.0294	0.4337
KTELC1	ILMN_1774800	0.0385	0.9367	696	0.0471	0.0107	136	0.0121	0.7699	96	-0.0243	0.6187
DDX19B	ILMN_2381841	0.0387	0.9367	718	-0.0344	0.1318	145	-0.0432	0.3283	96	-0.0557	0.1339
UBE2H	ILMN_1757644	0.0387	0.9367	718	0.0422	0.3071	152	0.1360	0.2015	96	0.1797	0.0143
KEAP1	ILMN_2410771	0.0388	0.9367	690	-0.0280	0.0737	105	-0.0279	0.3067	96	-0.0328	0.4785
TLR8	ILMN_1705047	0.0388	0.9367	718	0.0745	0.0167	148	0.0231	0.7827	96	-0.0034	0.9549
HSD17B11	ILMN_1735367	0.0389	0.9367	718	0.0568	0.0645	152	0.1578	0.0983	96	-0.0157	0.7677
TMEM217	ILMN_1756238	0.0390	0.9367	701	-0.0507	0.1151	135	0.0039	0.9493	96	-0.1213	0.0114
TRIP11	ILMN_1658144	0.0391	0.9367	709	-0.0470	0.0124	142	-0.0014	0.9624	96	0.0038	0.9256
MSH3	ILMN_2077130	0.0392	0.9367	718	0.0472	0.0417	152	-0.0371	0.4715	96	0.2265	0.0301
FAM8A1	ILMN_1807448	0.0392	0.9367	718	0.0625	0.0702	150	0.0588	0.5508	96	0.0732	0.2610
KRAS	ILMN_1728071	0.0393	0.9367	717	0.0402	0.0588	148	0.0105	0.8552	96	0.0641	0.1560
DDRGK1	ILMN_1797828	0.0395	0.9367	718	-0.0310	0.1128	152	-0.0661	0.2496	96	-0.0509	0.3020
FSTL4	ILMN_1661566	0.0396	0.9367	696	-0.0985	0.0185	139	-0.0579	0.5990	96	0.0142	0.7748
NUMB	ILMN_1705114	0.0396	0.9367	718	0.0395	0.1433	152	0.1416	0.0883	96	0.0379	0.5038
YIF1A	ILMN_1712975	0.0397	0.9367	717	-0.0499	0.0323	148	-0.0260	0.6594	96	-0.0200	0.6926
KIF22	ILMN_3234884	0.0397	0.9367	718	-0.0281	0.2140	151	-0.0921	0.0682	96	-0.0550	0.2624
MBIP	ILMN_1664323	0.0398	0.9367	707	0.0421	0.0264	82	0.0063	0.8238	96	0.0099	0.7732
ZNF302	ILMN_2307450	0.0401	0.9367	718	0.0959	0.0167	150	0.1126	0.3364	96	-0.0487	0.3397
ANG	ILMN_1760727	0.0402	0.9367	716	-0.0413	0.0877	148	-0.1037	0.0232	96	0.0412	0.4764
CCDC12	ILMN_1725071	0.0403	0.9367	718	-0.0370	0.0959	151	-0.1055	0.0528	96	0.0084	0.8427
ZDHHC6	ILMN_2046003	0.0404	0.9367	718	0.0516	0.0220	151	0.0728	0.2218	96	-0.0573	0.3117
CHKA	ILMN_1691151	0.0404	0.9367	712	-0.0834	0.0082	92	-0.0285	0.4507	96	0.0549	0.1658
DMTF1	ILMN_1750075	0.0405	0.9367	718	0.0712	0.0206	143	0.0995	0.2451	96	-0.0495	0.3188
GPBAR1	ILMN_2316386	0.0409	0.9367	718	-0.0571	0.1687	152	-0.2430	0.0258	96	-0.0127	0.8393
KCNK6	ILMN_1701173	0.0409	0.9367	716	-0.0448	0.0230	151	0.0037	0.9459	96	-0.0332	0.5255
LOC648024	ILMN_1665736	0.0409	0.9367	707	-0.0397	0.0571	139	0.0944	0.3091	96	-0.1433	0.0060
ANAPC11	ILMN_2343563	0.0409	0.9367	702	-0.0256	0.1029	124	-0.0443	0.0860	96	-0.0098	0.8013
FBP1	ILMN_1728799	0.0410	0.9367	717	-0.0334	0.2433	148	-0.1148	0.0756	96	-0.0816	0.1759
LOC100133465	ILMN_3243549	0.0411	0.9367	718	0.0244	0.1174	152	0.0106	0.8021	96	0.0827	0.0302
C10orf32	ILMN_1772706	0.0412	0.9367	695	-0.0165	0.1948	149	-0.1032	0.0975	96	-0.0573	0.2652
NENF	ILMN_2142554	0.0412	0.9367	718	-0.0542	0.0785	152	-0.0999	0.1838	96	-0.0152	0.7847
PDS5A	ILMN_1670931	0.0416	0.9367	718	0.0581	0.0128	150	0.0039	0.9568	96	-0.0068	0.8924
LOC389404	ILMN_3200661	0.0416	0.9367	718	0.1065	0.0204	152	0.1001	0.3373	96	-0.1041	0.4227
VENTX	ILMN_1782352	0.0417	0.9367	697	-0.0385	0.1095	139	-0.0966	0.1269	96	-0.0282	0.6354
LARP4B	ILMN_1766222	0.0418	0.9367	718	0.0440	0.0312	152	0.0447	0.3145	96	-0.0157	0.6800
NUDT2	ILMN_2349444	0.0418	0.9367	612	0.0469	0.0045	91	-0.0026	0.9446	96	-0.0446	0.3301
LOC729217	ILMN_3229467	0.0419	0.9367	715	0.0348	0.1070	136	0.1086	0.0148	96	-0.0356	0.5317
KIAA2010	ILMN_1803254	0.0420	0.9367	718	0.0620	0.0225	150	0.0490	0.4078	96	-0.0266	0.5840
AQR	ILMN_1717154	0.0424	0.9367	717	0.0362	0.0411	149	0.0173	0.6003	96	0.0221	0.6348
TLR8	ILMN_1657892	0.0425	0.9367	718	0.0605	0.0168	152	-0.0028	0.9738	96	0.0120	0.8225
ZADH2	ILMN_1795063	0.0426	0.9367	682	-0.0159	0.3199	134	-0.0562	0.1670	96	-0.0952	0.0252
NKTR	ILMN_2330994	0.0427	0.9367	710	0.0853	0.0264	79	0.0406	0.2775	96	-0.0356	0.5515

LOC728556	ILMN_1790819	0.0428	0.9367	718	0.0458	0.0235	151	0.0165	0.7732	96	0.0076	0.8808
C16orf13	ILMN_1774990	0.0428	0.9367	718	-0.0289	0.3415	149	-0.1236	0.0353	96	-0.0664	0.1409
TMEM55A	ILMN_1752117	0.0428	0.9367	708	0.0753	0.0465	146	0.1677	0.0891	96	-0.0444	0.3950
ZNF252	ILMN_3243961	0.0430	0.9367	717	0.0463	0.0436	148	0.0492	0.3060	96	-0.0042	0.9212
LOC728973	ILMN_1685378	0.0431	0.9367	718	0.0594	0.1157	152	0.2093	0.1559	96	0.0501	0.5400
TSPAN31	ILMN_1725079	0.0432	0.9367	718	-0.0158	0.4286	152	-0.1065	0.0292	96	-0.0850	0.0743
TDG	ILMN_1777096	0.0432	0.9367	718	0.0512	0.0297	151	0.0239	0.7785	96	0.0196	0.6931
MIF	ILMN_1807074	0.0432	0.9367	718	-0.0411	0.0657	152	-0.0743	0.2828	96	-0.0178	0.7582
FAM177A1	ILMN_2394438	0.0433	0.9367	718	0.0342	0.0428	152	-0.0206	0.6134	96	0.0855	0.0733
PARP9	ILMN_1731224	0.0434	0.9367	718	0.0785	0.0560	152	0.1131	0.2921	96	0.0100	0.8895
PRPF4B	ILMN_1736021	0.0435	0.9367	703	0.0728	0.0091	119	0.0378	0.4697	96	-0.0551	0.1805
TRAPPC2L	ILMN_1747058	0.0435	0.9367	718	-0.0418	0.1219	152	-0.1061	0.0635	96	-0.0065	0.9058
BRP44L	ILMN_1666967	0.0436	0.9367	698	0.0445	0.0175	134	0.0087	0.8173	96	-0.0062	0.8997
METTL13	ILMN_2343624	0.0436	0.9367	717	-0.0344	0.1113	151	-0.0810	0.0995	96	-0.0110	0.7976
LOC653737	ILMN_3213531	0.0436	0.9367	718	0.0279	0.2661	152	0.0930	0.2628	96	0.2156	0.0255
EDC4	ILMN_1665212	0.0436	0.9367	718	-0.0231	0.3881	152	-0.1713	0.0181	96	-0.0638	0.1782
MED23	ILMN_1690999	0.0436	0.9367	716	0.0796	0.0343	145	0.0354	0.6831	96	0.0186	0.7127
LOC727825	ILMN_1681325	0.0440	0.9367	713	-0.0249	0.0710	120	-0.0279	0.3453	96	-0.0237	0.5740
PTBP1	ILMN_1655154	0.0441	0.9367	718	-0.0296	0.1842	152	-0.0813	0.0709	96	-0.0338	0.4475
NOMO2	ILMN_1799856	0.0444	0.9367	718	-0.0350	0.2028	151	-0.1230	0.0624	96	-0.0518	0.4052
ZNF143	ILMN_1674399	0.0445	0.9367	718	0.0313	0.2032	152	0.1348	0.0114	96	-0.0005	0.9916
ABHD3	ILMN_1801767	0.0446	0.9367	715	0.0806	0.0485	103	0.1529	0.1365	96	-0.0285	0.6498
C20orf4	ILMN_1721225	0.0446	0.9367	716	-0.0300	0.1773	150	-0.0687	0.1114	96	-0.0460	0.3359
LIME1	ILMN_2183687	0.0446	0.9367	718	-0.0298	0.2106	152	-0.0826	0.3257	96	-0.1239	0.0467
NCF4	ILMN_1785005	0.0446	0.9367	718	0.0466	0.1194	152	0.0804	0.2727	96	0.0681	0.3111
TSHZ3	ILMN_1743933	0.0446	0.9367	711	-0.0525	0.0724	150	-0.0404	0.4068	96	-0.0345	0.4893
CLEC4D	ILMN_1808979	0.0447	0.9367	623	0.0425	0.0786	110	0.1174	0.0624	96	-0.0241	0.7529
LOC391370	ILMN_3293367	0.0447	0.9367	717	0.0125	0.5767	152	0.2427	0.0408	96	0.1573	0.0109
C21orf33	ILMN_1737588	0.0447	0.9367	718	-0.0354	0.0589	151	0.0223	0.6254	96	-0.0834	0.0368
GPR89B	ILMN_1804539	0.0448	0.9367	698	-0.0313	0.1702	143	-0.0426	0.3081	96	-0.0604	0.1092
COX8A	ILMN_1809495	0.0448	0.9367	718	-0.0367	0.0805	152	-0.0634	0.2302	96	-0.0159	0.7243
MRPL37	ILMN_2041327	0.0449	0.9367	718	-0.0403	0.0792	152	-0.0662	0.2674	96	-0.0201	0.6562
EIF2B2	ILMN_1713380	0.0449	0.9367	718	-0.0255	0.1670	150	-0.0490	0.1828	96	-0.0551	0.2344
AKTIP	ILMN_1665982	0.0449	0.9367	717	0.0463	0.0974	124	0.0582	0.2622	96	0.0298	0.4605
GAL3ST4	ILMN_1693452	0.0451	0.9367	712	0.0456	0.0274	148	-0.0048	0.9047	96	0.0308	0.4579
CAPZA2	ILMN_1768870	0.0452	0.9367	718	0.0599	0.0332	152	0.0831	0.2989	96	-0.0342	0.6192
RPL39	ILMN_1737015	0.0454	0.9367	718	0.0452	0.1557	152	0.0831	0.2390	96	0.1235	0.2064
SYPL1	ILMN_1764087	0.0454	0.9367	718	0.0477	0.0578	151	0.0546	0.4465	96	0.0242	0.6299
CTSC	ILMN_1696347	0.0454	0.9367	718	0.0529	0.0589	152	0.0257	0.7567	96	0.0599	0.2847
LOC644214	ILMN_3209117	0.0455	0.9367	603	0.0304	0.0092	116	0.0190	0.5243	96	-0.0561	0.2525
ADA	ILMN_1803686	0.0455	0.9367	717	-0.0230	0.4656	149	-0.1556	0.0076	96	-0.0581	0.1969
CBX1	ILMN_1770244	0.0456	0.9367	699	0.0299	0.1677	130	0.0480	0.2026	96	0.0738	0.2024

USP14	ILMN_1806804	0.0456	0.9367	678	-0.0228	0.1693	149	-0.0721	0.2652	96	-0.0684	0.1533
MED9	ILMN_1794108	0.0456	0.9367	685	-0.0213	0.0635	105	-0.0198	0.4191	96	-0.0231	0.5923
WDR55	ILMN_1678957	0.0457	0.9367	689	-0.0242	0.1111	131	-0.0174	0.6304	96	-0.0704	0.1069
ARF3	ILMN_1682938	0.0461	0.9367	717	-0.0568	0.0459	150	-0.0955	0.1791	96	0.0281	0.5891
ADRM1	ILMN_2389013	0.0462	0.9367	717	-0.0456	0.0493	151	0.0071	0.8797	96	-0.0637	0.1579
PPP2R1A	ILMN_1810467	0.0462	0.9367	718	-0.0375	0.0896	152	-0.0465	0.5440	96	-0.0571	0.2331
	ILMN_1821975	0.0463	0.9367	444	-0.0285	0.0995	91	-0.0022	0.9530	96	-0.1022	0.0834
LOC389787	ILMN_1665823	0.0463	0.9367	718	0.0316	0.2706	152	0.2699	0.0767	96	0.1213	0.1740
MCM5	ILMN_1815169	0.0464	0.9367	717	-0.0389	0.1649	146	-0.0349	0.3769	96	-0.0866	0.0920
C20orf196	ILMN_1793002	0.0464	0.9367	703	-0.0267	0.0744	86	-0.0360	0.1430	96	-0.0040	0.9225
LGALS8	ILMN_2356654	0.0466	0.9367	718	0.0569	0.0346	148	0.0726	0.2181	96	-0.0366	0.4661
PHKG2	ILMN_1669607	0.0467	0.9367	668	-0.0180	0.2466	107	-0.0712	0.0211	96	-0.0315	0.4469
FPR2	ILMN_2392569	0.0468	0.9367	712	0.0754	0.0766	125	0.1598	0.1234	96	-0.0094	0.9141
SF3B4	ILMN_1722648	0.0468	0.9367	718	-0.0408	0.0608	152	0.0031	0.9676	96	-0.0662	0.1316
PTP4A2	ILMN_2338997	0.0468	0.9367	718	-0.0378	0.0721	152	-0.0732	0.1523	96	0.0069	0.8914
C10orf73	ILMN_1738047	0.0468	0.9367	713	0.0569	0.0466	137	-0.0088	0.8699	96	0.0728	0.1975
BAZ1A	ILMN_1742230	0.0468	0.9367	718	0.0659	0.0330	152	0.0590	0.5523	96	0.0005	0.9938
DNAJB14	ILMN_2415898	0.0469	0.9367	711	0.0572	0.1657	147	0.1350	0.1023	96	0.0308	0.4544
CSNK1G1	ILMN_1704713	0.0472	0.9367	716	-0.0362	0.1220	151	-0.0780	0.1075	96	-0.0167	0.7498
C10orf118	ILMN_1734010	0.0472	0.9367	614	0.0203	0.0656	124	0.0375	0.2382	96	0.0047	0.9101
EOMES	ILMN_1760509	0.0472	0.9367	718	-0.0567	0.1243	151	-0.2251	0.0239	96	0.0384	0.6389
CLSTN1	ILMN_2415179	0.0472	0.9367	718	-0.0357	0.2705	152	-0.1056	0.2157	96	-0.0987	0.0456
UBL7	ILMN_1733991	0.0474	0.9367	718	-0.0815	0.0255	152	-0.0874	0.2877	96	0.0402	0.3775
EBPL	ILMN_1805922	0.0476	0.9367	717	0.0333	0.1292	136	0.0844	0.0377	96	-0.0061	0.9076
KBTBD7	ILMN_2044293	0.0476	0.9367	718	0.0668	0.0831	148	0.0447	0.4917	96	0.0588	0.3398
DRG2	ILMN_1810531	0.0476	0.9367	710	-0.0290	0.1324	120	-0.0259	0.3486	96	-0.0572	0.2007
MFAP3	ILMN_1708782	0.0477	0.9367	714	-0.0995	0.0163	116	0.0144	0.8595	96	-0.0029	0.9519
ZNF668	ILMN_1739236	0.0477	0.9367	701	-0.0136	0.3587	110	-0.0657	0.0266	96	-0.0586	0.1343
	ILMN_1877778	0.0478	0.9367	665	0.0136	0.1449	118	0.0342	0.2176	96	0.0461	0.2936
ZNF721	ILMN_1805271	0.0479	0.9367	716	0.0628	0.0664	145	0.0912	0.2440	96	0.0045	0.9394
PGLS	ILMN_1797005	0.0479	0.9367	718	-0.0322	0.2617	152	-0.1378	0.0831	96	-0.0604	0.1940
RAP1GDS1	ILMN_2106167	0.0482	0.9367	709	-0.0427	0.0508	111	-0.0210	0.5198	96	-0.0129	0.7121
FNTB	ILMN_1728517	0.0482	0.9367	706	-0.0504	0.0080	146	0.0081	0.8970	96	0.0304	0.5811
LOC729021	ILMN_1809173	0.0482	0.9367	718	0.0835	0.0265	152	0.0842	0.4482	96	-0.0413	0.6328
TLR10	ILMN_1719905	0.0483	0.9367	686	0.0398	0.0499	142	0.0768	0.1430	96	-0.0331	0.5315
SSU72	ILMN_1664956	0.0484	0.9367	718	-0.0317	0.0269	152	-0.0124	0.8009	96	-0.0074	0.8690
GPR137B	ILMN_2121816	0.0485	0.9367	712	-0.0621	0.0101	143	0.0440	0.3227	96	-0.0344	0.4972
CXCR4	ILMN_2320888	0.0485	0.9367	718	0.0584	0.0542	149	0.1632	0.1546	96	-0.0293	0.6074
TMEM189	ILMN_2162989	0.0485	0.9367	718	-0.0312	0.0949	151	-0.0653	0.1895	96	-0.0152	0.7531
PCCA	ILMN_1714384	0.0486	0.9367	711	-0.0174	0.1452	121	-0.0381	0.1208	96	-0.0228	0.4888
MMP23B	ILMN_2193325	0.0487	0.9367	711	-0.0371	0.2187	132	-0.0855	0.0204	96	-0.0183	0.7133
C20orf3	ILMN_1674394	0.0488	0.9367	718	0.0444	0.0569	152	0.0080	0.9130	96	0.0623	0.2374
ALOX12	ILMN_1713731	0.0488	0.9367	694	-0.0761	0.0404	138	-0.0329	0.6797	96	-0.0256	0.6979

SLC40A1	ILMN_2053103	0.0489	0.9367	718	0.0540	0.0982	152	0.1214	0.1616	96	0.0172	0.8110
MTX1	ILMN_1719403	0.0490	0.9367	694	-0.0141	0.1650	141	-0.0469	0.1683	96	-0.0543	0.3195
STRN3	ILMN_1772946	0.0491	0.9367	701	0.0445	0.0538	114	0.0503	0.3598	96	0.0044	0.9040
LOC100130291	ILMN_3190972	0.0493	0.9367	715	-0.0710	0.0040	106	0.0167	0.4758	96	0.0277	0.4500
PTGES2	ILMN_2345016	0.0493	0.9367	689	-0.0099	0.3151	108	-0.0541	0.0775	96	-0.0773	0.0902
CORO1B	ILMN_1653708	0.0494	0.9367	716	-0.0406	0.1294	149	-0.0914	0.1802	96	-0.0337	0.4982
DCTN1	ILMN_2412807	0.0495	0.9367	718	-0.0380	0.2491	152	-0.1659	0.0787	96	-0.0542	0.2552
FLJ31306	ILMN_3189585	0.0497	0.9367	718	0.0725	0.0281	152	0.0228	0.8393	96	0.0142	0.8086
HSPC047	ILMN_1726678	0.0497	0.9367	706	-0.0272	0.0448	93	-0.0035	0.8892	96	-0.0255	0.4989
TPR	ILMN_1730999	0.0498	0.9367	718	0.0386	0.1038	152	0.0837	0.1774	96	0.0187	0.7241
LOC285550	ILMN_1675803	0.0498	0.9367	710	0.0644	0.0165	139	-0.0024	0.9500	96	-0.0009	0.9842
MNT	ILMN_1792910	0.0498	0.9367	717	-0.0366	0.2079	151	-0.0885	0.3255	96	-0.0895	0.0699
LOC401115	ILMN_1668629	0.0498	0.9367	718	-0.0322	0.1793	152	-0.1252	0.0669	96	-0.0290	0.6064
